# Formation of metallacarboxylic acids through Hieber base reaction. A density functional theory study

**DOI:** 10.1007/s00894-018-3915-1

**Published:** 2019-01-25

**Authors:** Shahbaz Ahmad, Elisabeth A. Berry, Conor H. Boyle, Christopher G. Hudson, Oliver W. Ireland, Emily A. Thompson, Michael Bühl

**Affiliations:** School of Chemistry, University of St Andrews, North Haugh, St Andrews, Fife, KY16 9ST UK

**Keywords:** Homogeneous catalysis, Water gas shift reaction, Hieber base reaction, Density functional theory

## Abstract

Using density functional theory (B97-D/ECP2/PCM//RI-BP86/ECP1 level), we have studied the effects of ligand variation on OH^−^ uptake by transition-metal carbonyls (Hieber base reaction), i.e., L_*n*_M(CO) + OH^−^ → [L_*n*_M(CO_2_H)]^−^, M = Fe, Ru, Os, L = CO, PMe_3_, PF_3_, py, bipy, Cl, H. The viability of this step depends notably on the nature of the co-ligands, and a large span of driving forces is predicted, ranging from ΔG = −144 kJ/mol to +122 kJ/mol. Based on evaluation of atomic charges from natural population analysis, it is the ability of the co-ligands to delocalize the additional negative charge (through their π-acidity) that is the key factor affecting the driving force for OH^−^ uptake. Implications for the design of new catalysts for water gas shift reaction are discussed.

**Electronic supplementary material:**

The online version of this article (10.1007/s00894-018-3915-1) contains supplementary material, which is available to authorized users.

**Graphical abstract:**
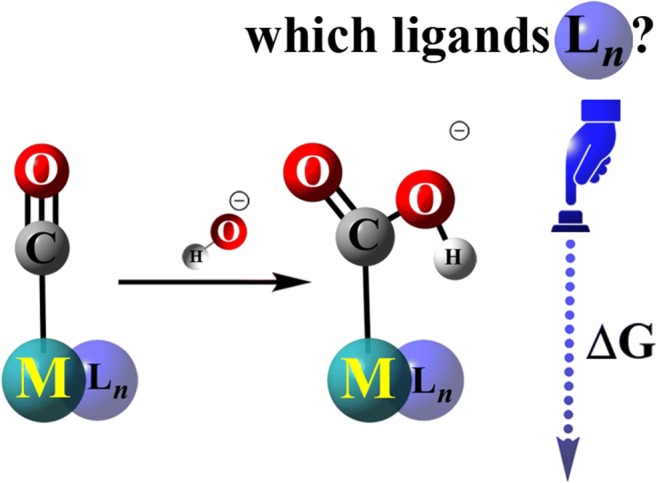
ᅟ

## Introduction

**Scheme 1:**
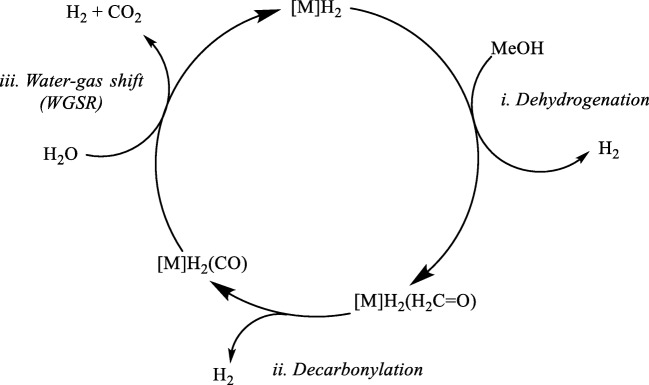
Putative reaction sequence for complete methanol dehydrogenation

Hydrogen is the cleanest fuel and a cost-effective energy carrier of the future [1–6], which produces three times more energy per unit mass than fossil fuels [7]. Industrially, hydrogen is generated from fossil fuels releasing higher amounts of greenhouse gas, CO_2_ [8, 9]. A sustainable supply of hydrogen from renewable resources, such as biomass, is highly desirable [4, 10]. Development of homogeneous transition metal catalysts for complete decomposition of polyhydroxy biomass constituents, i.e., carbohydrates, into H_2_ and CO_2_, could revolutionize H_2_ production from renewable resources. Methanol is the simplest model for such carbohydrates, but complete dehydrogenation of this compound according to

1 $$ \mathrm{MeOH}+{\mathrm{H}}_2\mathrm{O}\to {3\mathrm{H}}_2+{\mathrm{CO}}_2 $$

would be of considerable interest in its own right. This decomposition of methanol could follow a sequence of three reactions, (i) dehydrogenation, (ii) decarbonylation, and (iii) water–gas shift reaction (WGSR, see Scheme 1), all of which are well known.

Development of homogeneous transition metal catalysts for H_2_ production from methanol [11, 12] has attracted much attention in the past few decades. Morton and Cole-Hamilton developed a ruthenium catalyst [Ru(H)_2_(X_2_)(PPh_3_)_3_] (X = N, H) for partial dehydrogenation of alcohols (including methanol) with notable turnover frequencies [13]. Aldehydes and ketones were the main products [formaldehyde in case of methanol, Scheme 1, step (i)]. During the conversion of ethanol, significant amounts of methane and a carbonyl complex, [RuH_2_(CO)(PPh_3_)_3_], were produced through decarbonylation [Scheme 1, step (ii)]. However, since no CO_2_ was noticed, apparently these Ru complexes are not active as WGSR catalysts [14].

**Scheme 2:**
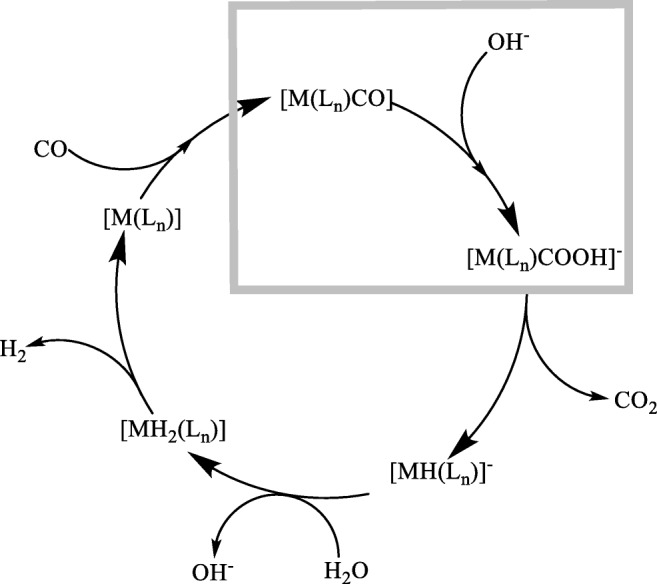
General mechanism for transition-metal-catalyzed WGSR under basic conditions, where OH^−^ is the nucleophile

Recently, based on density functional theory (DFT) calculations, we studied the mechanisms for the dehydrogenation [15] and decarbonylation [16] of aliphatic alcohols catalyzed by the Morton and Cole-Hamilton system, [RuH_2_(H_2_)(PPh_3_)_3_] [Scheme 1, steps (i) and (ii)]. When exploring the feasibility of WGSR with these complexes computationally [17], we noticed that the first step of WGSR, the attack of water (in the form of OH^−^) on the CO ligand (Scheme 2) is highly endergonic.

Significant research has been undertaken to reveal the mechanism of WGSRs catalyzed by homogeneous transition metal complexes, particularly the metal carbonyls of Fe, Ru, and Os [18–30]. Recently, Guo et al. have studied the WGSR mechanism catalyzed by hexacarbonyl complexes of Mo and W [31]. In all of these reactions, which are conducted under basic conditions, OH^−^ is the nucleophile and its uptake to form a transient metallacarboxylic acid is considered as the initial step (in the grey box in Scheme 2). Such attack of OH^−^ on carbonyl ligands is well known as Hieber base reaction [32].

In all studies of metal carbonyl catalyzed WGSR, this OH^−^ uptake appeared to be highly exothermic and essentially barrierless. In contrast, in our study of WGSR in the Morton and Cole-Hamilton system [17], this step is predicted to be highly endergonic. The driving force for formation of the metallacarboxylic acid depends notably on the co-ligands that are present, in particular on the number of CO ligands. While large negative enthalpies and free energies are computed for the OH^−^ uptake of Ru(CO)_5_ [28, 29] ΔG = 127.7 kJ/mol and 81.6 kJ/mol are predicted for [RuH_2_(CO)(PPh_3_)_3_] and [RuH_2_(CO)_2_(PPh_3_)_2_], respectively. It therefore appears that suitable ligand design, by varying the steric or electronic properties of the ligands, could make the process of OH^−^ uptake feasible. In this work, we now report DFT-computed driving forces for OH^−^ uptake in a number of metal-carbonyl complexes of Ru, Fe, and Os. Along with CO, we have made the choice of trimethylphosphine (comparable to triphenylphosphine), trifluorophosphine (a strong π-acceptor ligand, comparable to CO) [33], pyridine, and bipyridine ligands. For a perfect catalytic system, the OH^−^ entry into the cycle should be facile and should not produce a very low-lying intermediate on the reaction profile that would eventually deactivate the catalytic system. This work can lead to the rational design of better catalysts for WGSR and, eventually, towards the complete decomposition of alcohols by dehydrogenation, decarbonylation, and the finally WGSR, which could facilitate entry into a hydrogen-based economy.

## Results and discussion

### Ligand effects on the initial uptake of OH^−^ to form the metallacarboxylic acid

In metal carbonyls, OH^−^ uptake is usually considered a fast process, and the resulting metallacarboxylic acid is usually too reactive to be isolated and decarboxylates under CO_2_ evolution to form a hydride (Scheme 2). Protonation of this hydride intermediate takes the system uphill on the free energy reaction profile and the H_2_ evolution tends to be associated with the highest energy transition state. Our work focuses on the ligand effects on the initial uptake of OH^−^ to metal carbonyls (Eq. 2, corresponding to the first step in the grey box in Scheme 2), taking M(CO)_5_ pentacarbonyls as prototypical representatives (M = Fe, Ru, Os). Step by step, we replaced each of the CO in the metal pentacarbonyl system with selected ligands, namely trimethylphosphine (PMe_3_), trifluorophosphine (PF_3_), pyridine (py), and bipyridine (bipy). We compared the free energies of the reactants (metal carbonyls) and the products (metallacarboxylic acids) to see how such a ligand change affects the driving force for the OH- uptake to the system. We have included the results for Ru carbonyls and metallacarboxylic acids in the main paper, whereas the results for the Fe and Os analogs are included within the supporting information (SI).

2 $$ {\mathrm{L}}_n\mathrm{M}\left(\mathrm{CO}\right)+{\mathrm{OH}}^{-}\to {\left[{\mathrm{L}}_{\mathrm{n}}\mathrm{M}\left({\mathrm{CO}}_2\mathrm{H}\right)\right]}^{-},\mathrm{M}=\mathrm{Fe},\mathrm{Ru},\mathrm{Os},\mathrm{L}=\mathrm{CO},{\mathrm{PMe}}_3,{\mathrm{PF}}_3,\mathrm{py},\mathrm{bipy},\mathrm{Cl},\mathrm{H} $$

**Fig. 1:**
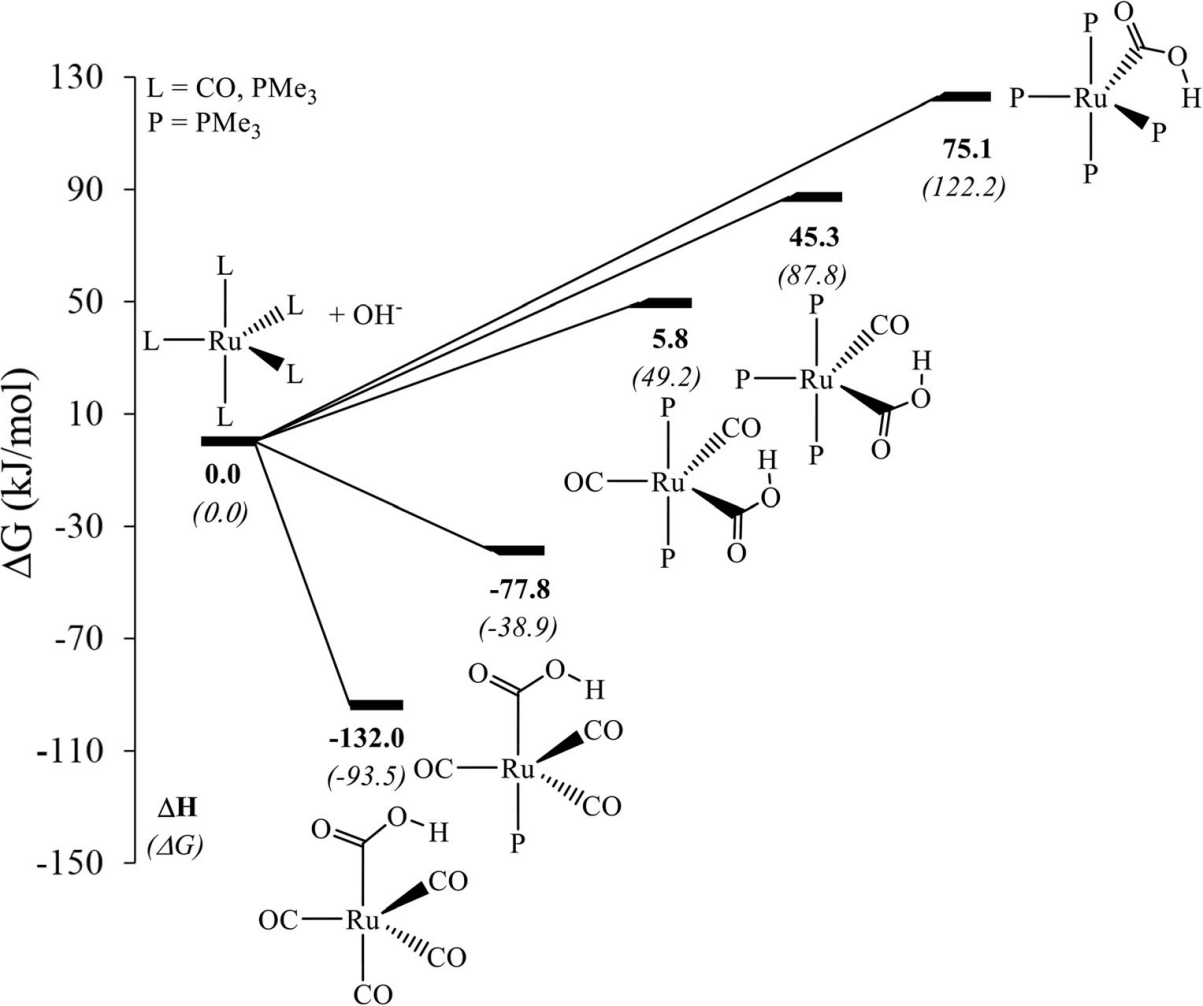
Computed free energies (B97-D level, kJ/mol) for the OH^−^ uptake of the carbonyl reactant (note that this reactant is different for each product). The number of PMe_3_ ligands increases from left to right (see Fig. S1 in the ESI for a plot showing three-dimensional representations of the complexes)

Unlike CO, PMe_3_ is a weak π-acceptor ligand and experiences weak backbonding with the metal center. At our chosen level of theory, B97-D/ECP2//RI-BP86/ECP1, Ru(CO)_5_ has a driving force of ΔG = −93.5 kJ/mol for the initial OH^−^ uptake. This driving force decreases (i.e., ΔG increases) as we increase the number of PMe_3_ ligands that replace CO. On substituting one CO with one PMe_3_ ligand at the axial position, the free energy increases to −38.9 kJ/mol, which further increases to 49.2 kJ/mol on replacing the second CO on the axial position with another PMe_3_ ligand [34]. The OH^−^ uptake to Ru(CO)_2_(PMe_3_)_3_ is unfavorable by a free energy of 87.8 kJ/mol, that of Ru(CO)(PMe_3_)_4_ by 122.2 kJ/mol (Fig. 1). Since the subsequent steps on the WGSR reaction profile (Scheme 2) add additional barriers, the latter two complexes are expected to be only weakly active or unproductive as WGSR catalysts.

**Fig. 2:**
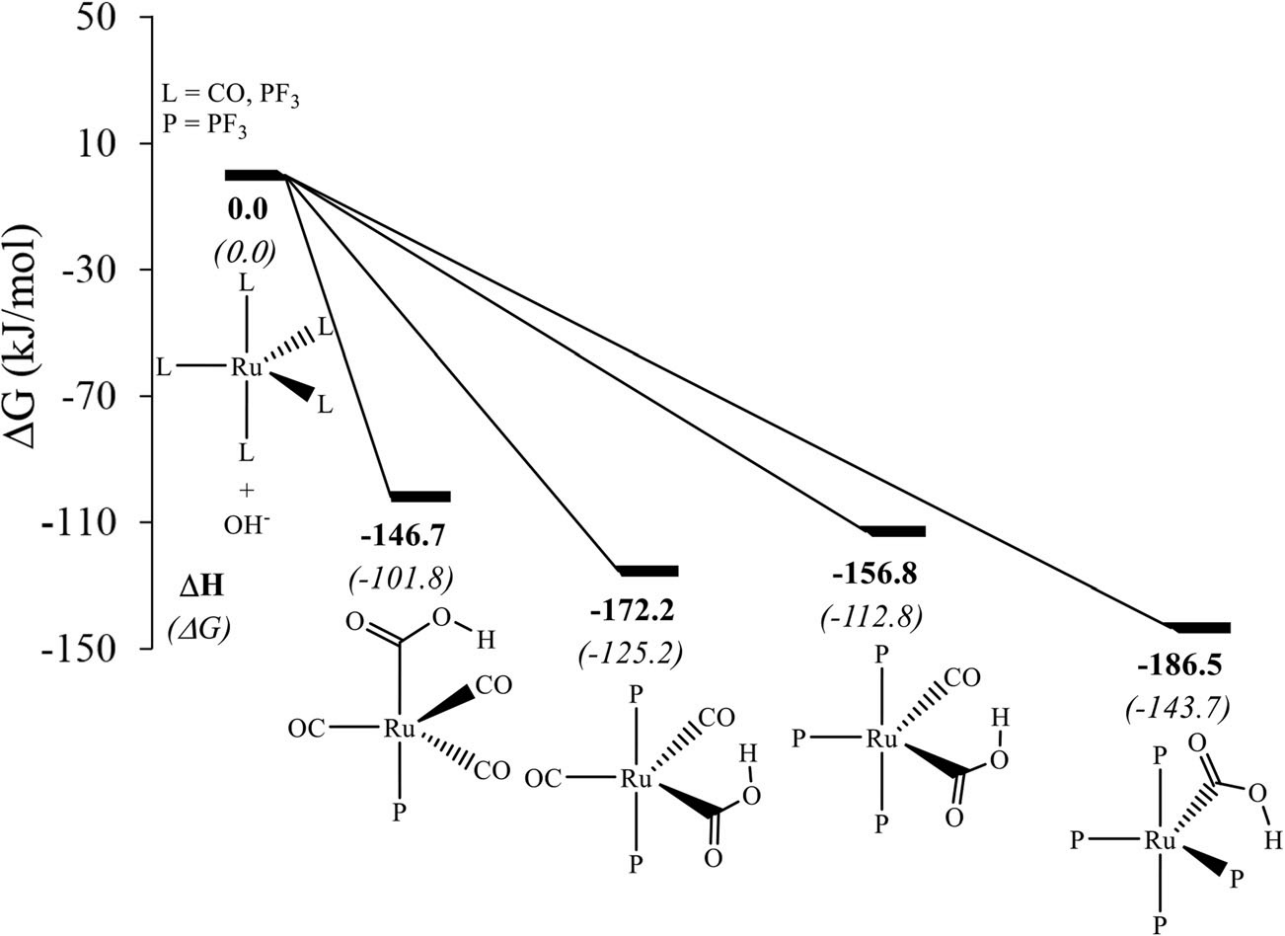
Relative free energy (kJ/mol) for the OH^−^ uptake with that of the respective carbonyl reactant set to 0.0 kJ/mol in each case. The number of PF_3_ ligands increases from left to right (see Fig. S2 in the ESI for a plot showing three-dimensional representations of the complexes)

Based on the results obtained for the PMe_3_ ligand exchange with CO, one can assume that the driving force for the OH^−^ uptake should be affected by replacing CO ligands with ligands of slightly greater π-acceptor strength, e.g., PF_3_. A slight increase of the driving force for OH^−^ uptake is observed on replacing one CO at the axial position with a PF_3_ ligand, from −93.5 kJ/mol in Ru(CO)_5_ (Fig. 1) to −101.8 kJ/mol for Ru(CO)_4_(PF_3_) (Fig. 2). On exchanging both axial CO ligands with PF_3_, the free energy decreases by 31.7 kJ/mol for the OH^−^ uptake as compared to the free energy of the OH^−^ uptake in Ru(CO)_5_. One would expect that exchanging three CO ligands, two at the axial and one at the equatorial position, should further favor OH^−^ uptake, but this is not the case. After the OH^−^ uptake by Ru(CO)_2_(PF_3_)_3_, the metalla-acid, [Ru(CO)(COOH)(PF_3_)_3_]^−^, is obtained with a free energy of −112.8 kJ/mol, which is higher by 12.5 kJ/mol than the free energy for the OH^−^ uptake in Ru(CO)_2_(PF_3_)_3_, possibly because of the *trans* influence of CO. When employing another, higher-lying isomer of Ru(CO)_2_(PF_3_)_3_, where one of the CO ligands is positioned *trans* to PF_3_, the resulting metalla-acid is obtained at a ΔG of −121.4 kJ/mol. This value is lower by 8.6 kJ/mol compared to the ΔG for the most stable isomer included in Fig. 2. There is thus a noticeable *trans* influence on the driving force under scrutiny, although it does not seem to override other electronic effects.

Ru(CO)(PF_3_)_4_ follows the expected trend in terms of free energy for the OH^−^ uptake and has the largest predicted affinity for OH^−^ of all complexes studied here (−143.7 kJ/mol, Fig. 2). It should be noted that such a large driving force for OH^−^ uptake does not necessarily make this complex a good target for a WGSR catalyst, because a correspondingly higher energy needs to be invested to close the cycle and re-form the initial catalyst.

**Fig. 3:**
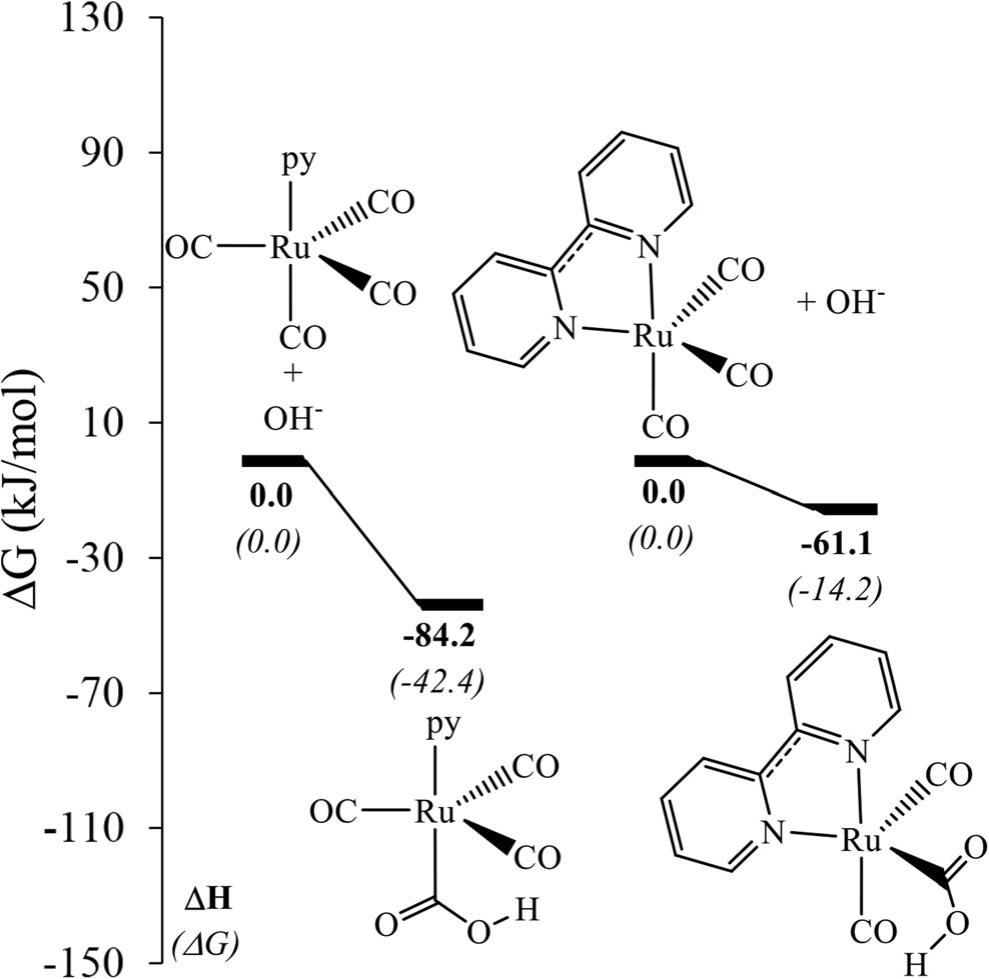
Relative free energy (kJ/mol) for the OH^−^ uptake on replacing one CO ligand with py (*left*) and two CO ligands with bipy (*right*) (see Fig. S3 in the ESI for a plot showing three-dimensional representations of the complexes)

A variety of [Ru]-CO_2_H complexes are known, some of which have been structurally characterized, notably with bidentate aromatic N-donor ligands [35, 36]. We therefore included a couple of model complexes with aromatic N-donor ligands, namely pyridine (py) and bipy. Both are coordinated through the lone pair of an electronegative N atom providing inductive donation, with the aromatic backbone allowing for significant π-backbonding interaction. As the inductive donation from the nitrogen lone pair is counteracted by the backbonding into the aromatic system, on exchanging one CO at the axial position in Ru(CO)_5_ with a pyridine (py), the OH^−^ uptake becomes unfavorable as compared to that in parent Ru(CO)_5_, but not by that much as in case of PMe_3_ ligand. On replacing two CO with a bipy bidentate ligand, the OH^−^ uptake becomes further unfavorable as compared to that in parent Ru(CO)_5_, but remains favorable by 63.4 kJ/mol as compared to the OH^−^ uptake in the Ru(CO)_3_(PMe_3_)_2_ system (Fig. 3).

**Fig. 4:**
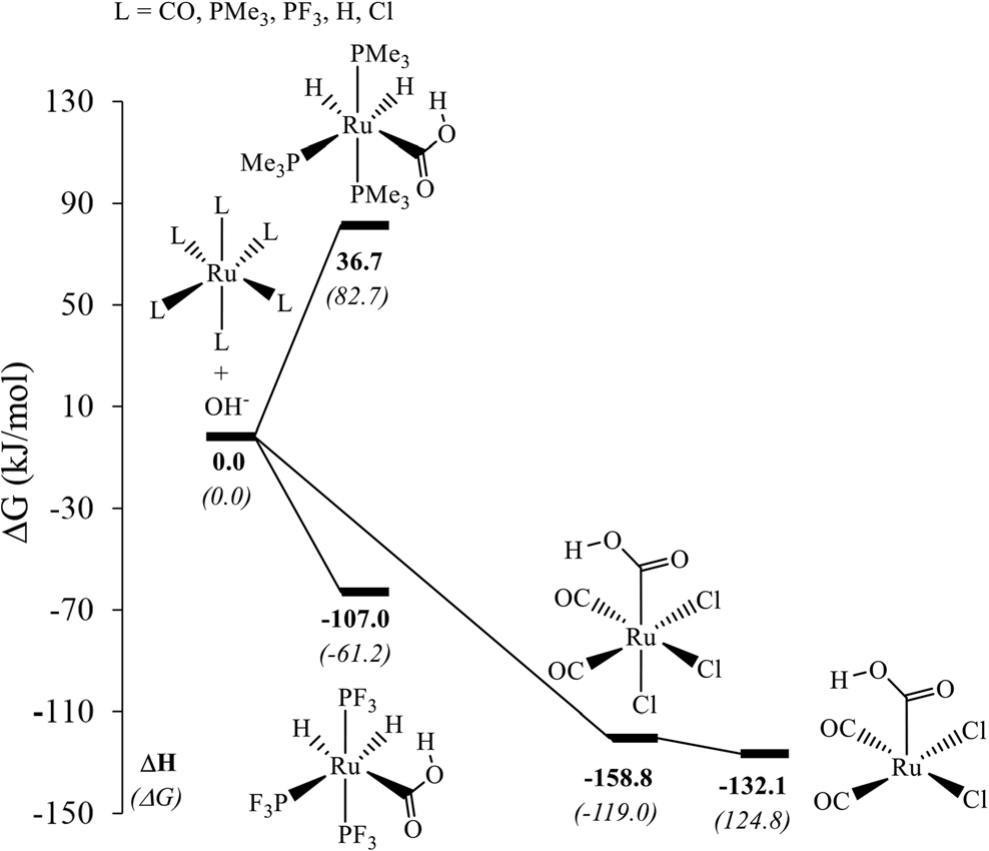
Relative free energy (kJ/mol) for the OH^−^ uptake with that of the respective octahedral carbonyl reactant set to 0.0 kJ/mol in each case (see Fig. S4 in the ESI for a plot showing three-dimensional representations of the complexes)

Finally, in addition to the pentacoordinate Ru(0) species, we considered the OH^−^ uptake in a few selected octahedral Ru(II) complexes including Ru(CO)(H)_2_(PMe_3_)_3_, Ru(CO)(H)_2_(PF_3_)_3_, and [Ru(CO)_3_Cl_3_]^−^ (Fig. 4). The driving force for OH^−^ uptake in Ru(CO)(H)_2_(PMe_3_)_3_, endergonic by 82.7 kJ/mol, is comparable to that in the Morton and Cole-Hamilton system, Ru(CO)(H)_2_(PPh_3_)_3_, where it is endergonic by 127 kJ/mol at essentially the same level (at a higher temperature, however, 150 °C) [17, 37]. On replacing PMe_3_ ligands with PF_3_ ligands, the product is obtained at a free energy of −61.2 kJ/mol, obviously because of strong π-backbonding interaction.

The results for the analogous Os complexes are very similar to those for the Ru species just discussed, with individual driving forces for OH^−^ uptake within typically 10 kJ/mol of each other (ca. 20 kJ/mol for the bipy complex, compare Tables S1 and S3 in the SI). On going from Ru to Fe congeners, the changes in this driving force become somewhat more variable (up to ca. 30 kJ/mol, compare Tables S1 and S2 in the SI), but overall the same trends are obtained irrespective of the group 8 metal.

The metallacarboxylic acid arising from Hieber base reaction of [Ru(CO)_3_Cl_3_]^−^ has been implicated as a key reactive intermediate in a complex variety of reactions [32]. Indeed, despite forming a dianion from two monoions, OH^−^ uptake of [Ru(CO)_3_Cl_3_]^−^ affording [Ru(CO)_2_(CO_2_H)Cl_3_]^2−^ is highly exergonic, with a free energy of −119.0 kJ/mol. This large driving force is fully consistent with the fact that this complex is a reactive intermediate that can be formed through Hieber base reaction [32]. Experimentally, [Ru(CO)_2_(CO_2_H)Cl_3_]^2−^ appears to lose a chloride ion consistent with our calculations as at our level, as this process is computed to be slightly exergonic, by −5.9 kJ/mol.

### Natural population analysis

What is the origin of the huge variation in driving forces for OH^−^ uptake in these complexes? Hypothesizing that a key factor should be delocalization of the additional negative charge brought into the complex, we used natural population analysis (NPA) [38] to evaluate the extent of charge transfer from OH^−^ upon attack on the carbonyl ligand. To this end, we simply calculated the natural charge on the OH^−^ fragments in the ruthenacarboxylic acid products, assessing how it changes from the value in free OH^−^, where it is −1. A substantial reduction from this absolute value is found in the complexes, indicating that most of the charge is actually delocalized into the complex, but there is still a notable variation of this charge, between −0.32 and −0.19 (see Table S4 in the SI).

The PMe_3_ ligand has σ-donating abilities, which pushes electron density to the metal center, which increases the amount of backbonding interaction between the filled metal *d*-orbital and the empty π*- orbital of the carbon atom of CO. The overall affect makes it difficult for the OH^−^ fragment to delocalize electron density over the metal complex. As we replace more CO ligands with PMe_3_ ligands, the natural charge at the OH^−^ fragment decreases. In [Ru(CO)_2_(CO_2_H)(PMe_3_)_2_]^−^, presence of a CO ligand at the axial position *trans* to the –CO_2_H^−^ fragment increases its distance from the metal center, making it less available for OH^−^ fragment to accommodate the charge density. Here the *trans* influence dominates the electronic nature of the ligands and a small discrepancy in the natural charges of the OH^−^ fragment occurs when we move from [Ru(CO)_2_(CO_2_H)(PMe_3_)_2_]^−^ to [Ru(CO)(CO_2_H)(PMe_3_)_3_]^−^, similar is the case with py and bipy ligands. For the PF_3_ ligands, on replacing each with the CO ligands, the natural charge at the OH^−^ fragment gradually increases, as expected (Table S4).

**Fig. 5:**
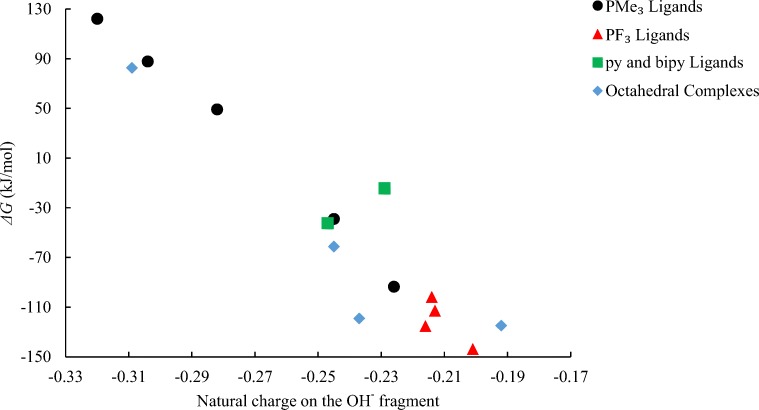
Plot of driving forces for OH^−^ uptake vs. natural charges of the OH^−^ fragments in the products (B97-D level)

A plot of the computed driving forces vs. OH^−^ charges indeed reveals an overall trend towards more favorable OH^−^ uptake with decreasing charge on this fragment (see Fig. 5; essentially the same correlation is obtained when enthalpies are used instead of free energies, see Fig. S5 in the SI). No strict relationship is apparent, and there are a few outliers, notably the bipy complex, but overall our results are consistent with the charge delocalization being a key factor for the driving force of this reaction step. For instance, the PF_3_ ligands, which are predicted to strongly promote Hieber base reaction (Fig. 2), are indicated to do so because they are very efficient in delocalizing the incoming negative charge (see red triangles in the lower right of Fig. 5). Arguably, the extent of this charge delocalization will depend on the balance between σ-donating and π-backdonating capabilities of the co-ligands, which should allow for a rational tailoring of complexes toward Hieber base reaction and, eventually, for designing new WGSR catalysts.

## Conclusions

In summary, using an appropriate DFT level, we have computed the driving forces for formation of metallacarboxylic acids from group 8 carbonyl complexes through uptake of OH^−^. This reaction (Eq. 2), known as Hieber base reaction, is the first step of water–gas shift reaction (WGSR) that can be catalyzed by transition metal complexes under basic conditions. According to our findings, the driving force for this step is surprisingly sensitive to the nature of the co-ligands at the metal, and can range from ΔG = −144 kJ/mol to +122 kJ/mol [for R = F and Me, respectively, in Ru(CO)(PR_3_)_4_]. Far from being innocent spectator ligands, these co-ligands actively take part in OH^−^ uptake through delocalization of the negative charge, as apparent in the computed atomic charges from natural population analysis. Fe and Ru pentacarbonyls are prototypical WGSR catalysts; it is remarkable how replacement of CO ligands with electron-rich phosphines (which are ubiquitous in modern transition metal chemistry) can impede the first step of this WGSR catalytic cycle. In that case, use of phosphines with electron-withdrawing substituents (where we have used PF_3_ as extreme example) or aromatic N-donor ligands can increase the driving force for Hieber base reaction. Compared to these ligand effects, the nature of the metal (Fe, Ru, or Os) or its oxidation state [e.g., Ru(0) vs. Ru(II)] seems to be of lesser importance for OH^−^ uptake.

We are convinced that the tunability of the driving force for Hieber base reaction through appropriate choice of co-ligands can inform on the rational design of new WGSR catalysts. As this quantity, a simple reaction (free) energy, can be readily computed with modern DFT tools, large libraries of ligands can be screened computationally, opening up new avenues for applications of molecular modeling in homogeneous catalysis.

## Computational methodology

In this work, we are mainly interested in calculating the driving force related to change in Gibbs free energy for the OH^−^ uptake, which is considered as the initial step of the WGSR. Our calculations follow a computationally cost-effective protocol based on density functional theory (DFT) that had been validated [39] and fruitfully applied to mechanistic DFT studies of related Ru complexes [15, 16]. Geometry optimizations were carried out at a lower level, RI-BP86/ECP1, whereas the energies were refined at B97-D/ECP2 [40] level. All the metal complexes were fully optimized at the RI-BP86/ECP1 level, i.e., by the use of Becke [41] and Perdew [42] exchange and correlation functionals along with SDD [43] core potential and valence basis on the metal atoms, whereas all the other atoms were treated with the standard 6-31G(d,p) basis. The nature of all the minima was verified by frequency calculations within the harmonic approximation, which were further used to obtain the enthalpic and entropic corrections under standard conditions (1 atm and 298.15 K). Thermochemical correction terms *δE*_*G*_ were obtained as:

3 $$ \delta {E}_G=\varDelta {G}_{RI- BP86/ ECP1}-\varDelta {E}_{RI- BP86/ ECP1} $$

where *ΔE*_*RI-BP86/ECP1*_ is the reaction energy and *ΔG*_*RI-BP86/ECP1*_ is the corresponding Gibbs free energy (analogously for corrections to enthaly, *δE*_*H*_ from *ΔH*_*RI-BP86/ECP1*_ - *ΔE*_*RI-BP86/ECP1*_).

The energies of the optimized complexes were refined through single-point calculations at the B97-D/ECP2 level, i.e., using dispersion-corrected B97-D functional, which includes Grimme’s dispersion correction [40] along with 6-311+G(d,p) basis set for all the non-metal atoms (SDD on the metals). Solvent effects were included by a polarizable continuum model (PCM) [44, 45] using methanol as a model solvent with self-consistent reaction field (SCRF) method. The solvent energy correction, *δE*_solv_, was performed as:

4 $$ \delta {E}_{solv}=\varDelta {E}_{PCM}-\varDelta E $$

Difference between the reaction energy in the continuum is donated as *ΔE*_*PCM*_ and *ΔE* is the difference between the reaction energy in gas phase at B97-D/ECP2 level. The final enthalpies and free energies, *ΔH* and *ΔG*, were obtained as sum of all energy correction terms:

5a $$ \varDelta H=\varDelta E+\delta {E}_{solv}+\delta {E}_H $$

5b $$ \varDelta G=\varDelta E+\delta {E}_{solv}+\delta {E}_G $$

where *ΔE* and *δE*_solv_ were calculated at the B97-D/ECP2 level, whereas *δE*_H_ and *δE*_G_ were obtained at RI-BP86/ECP1. Atomic charges from natural population analysis [38] were evaluated at the B97-D/ECP2/PCM level. All calculations were performed using the Gaussian 09 suite of programs [46].

In order to identify the most stable isomers and conformers of each reactant and product, an exhaustive screening of the possible stereoisomers was undertaken. Only the results for the most stable forms are reported. The conformation of the carboxylic acid group was uniformly taken as that where the hydrogen of the OH^−^ fragment is pointing towards the metal center. We investigated the stability of these metalla-acids against those in which the hydrogen of the OH^−^ fragments points away from the metal center, particularly in Fe complexes, the former complexes are appeared to be more stable (see Table S5).

## Electronic supplementary material

ESM 1 (PDF 1045 kb)
