# Supplementary material for: Formation of metallacarboxylic acids through Hieber base reaction. A density functional theory study
Source: J Mol Model. 2019 Jan 25;25(2):45. doi: 10.1007/s00894-018-3915-1 (PMC6347588; doi:10.1007/s00894-018-3915-1)
Supplement: Supplementary file 1 — (PDF 1045 kb) [file 894_2018_3915_MOESM1_ESM.pdf]

Supporting Information for

Formation of metallacarboxylic acids through Hieber base reaction. A density functional theory study.

Shahbaz Ahmad, Elisabeth A. Berry, Conor H. Boyle, Christopher G. Hudson, Oliver W. Ireland, Emily A. Thompson, Michael Bühl*

School of Chemistry, University of St Andrews, North Haugh, St Andrews, Fife KY16 9ST, United Kingdom, email: buehl@st-andrews.ac.uk

Full reference: Gaussian 09, Revision D.01, M. J. Frisch, G. W. Trucks, H. B. Schlegel, G. E. Scuseria, M. A. Robb, J. R. Cheeseman, G. Scalmani, V. Barone, B. Mennucci, G. A. Petersson, H. Nakatsuji, M. Caricato, X. Li, H. P. Hratchian, A. F. Izmaylov, J. Bloino, G. Zheng, J. L. Sonnenberg, M. Hada, M. Ehara, K. Toyota, R. Fukuda, J. Hasegawa, M. Ishida, T. Nakajima, Y. Honda, O. Kitao, H. Nakai, T. Vreven, J. A. Montgomery, Jr., J. E. Peralta, F. Ogliaro, M. Bearpark, J. J. Heyd, E. Brothers, K. N. Kudin, V. N. Staroverov, T. Keith, R. Kobayashi, J. Normand, K. Raghavachari, A. Rendell, J. C. Burant, S. S. Iyengar, J. Tomasi, M. Cossi, N. Rega, J. M. Millam,

M. Klene, J. E. Knox, J. B. Cross, V. Bakken, C. Adamo, J. Jaramillo, R. Gomperts, R. E. Stratmann, O. Yazyev, A. J. Austin, R. Cammi, C. Pomelli, J. W. Ochterski, R. L. Martin, K. Morokuma, V. G. Zakrzewski, G. A. Voth, P. Salvador, J. J. Dannenberg, S. Dapprich, A. D. Daniels, O. Farkas, J. B. Foresman, J. V. Ortiz, J. Cioslowski, and D. J. Fox, Gaussian, Inc., Wallingford CT, **2013**.

Table S1. Reaction Energies in gas phase (***ΔE***ΔE_{GAS})[a]; Solvation (δE_{solv})[a,b] and Thermochemistry (δE_G and δE_H)[c]; Final Free Energies (***ΔG***) in kJ/mol and Enthalpies (***ΔH***) for the OH^- uptake to the carbonyl complexes of ruthenium.

Reaction	ΔE_{GAS}	δE_{solv}	δE_G	δE_H	ΔG	ΔH
$RuCO_5 + OH^- \rightarrow [Ru(CO)_4(CO_2H)]^-$	-317.5	172.7	51.4	12.9	-93.5	-132.0
PMe₃ Ligands						
$Ru(CO)_4(PMe_3) + OH^- \rightarrow$ [Ru(CO)3(CO2H)(PMe3)]$^-$	-244.1	155.0	50.2	11.3	-38.9	-77.8
$Ru(CO)_3(PMe_3)_2 + OH^- \rightarrow [Ru(CO)_2(CO_2H)(PMe_3)_2]^-$	-166.6	163.2	52.5	9.1	49.2	5.8
$Ru(CO)_2(PMe_3)_3 + OH^- \rightarrow [Ru(CO)(CO_2H)(PMe_3)_3]^-$	-151.9	191.2	48.5	6.1	87.8	45.3
$Ru(CO)(PMe_3)_4 + OH^- \rightarrow [Ru(CO_2H)(PMe_3)_4]^-$	-120.5	190.2	52.5	5.3	122.2	75.1
PF₃ Ligands						
$Ru(CO)_4(PF_3) + OH^- \rightarrow [Ru(CO)_3(CO_2H)(PF_3)]^-$	-341.3	182.0	57.5	12.5	-101.8	-146.7
$Ru(CO)_3(PF_3)_2 + OH^- \rightarrow [Ru(CO)_2(CO_2H)(PF_3)_2]^-$	-374.1	189.6	59.3	12.3	-125.2	-172.2
$Ru(CO)_2$(PF3)$_3 + OH^- \rightarrow [Ru(CO)(CO_2H)(PF_3)_3]^-$	-368.2	200.2	55.2	11.2	-112.8	-156.8
$Ru(CO)(PF_3)_4 + OH^- \rightarrow [Ru(CO_2H)(PF_3)_4]^-$	-399.5	201.6	54.1	11.3	-143.7	-186.5
py and bipy Ligands						
$Ru(CO)_4(py) + OH^- \rightarrow [Ru(CO)_3(CO_2H)(py)]^-$	-241.6	146.4	52.8	11.0	-42.4	-84.2
$Ru(CO)_3(bipy) + OH^- \rightarrow Ru(CO)_2(CO_2H)(bipy)]^-$	-253.0	182.4	56.5	9.5	-14.2	-61.1
Octahedral Complexes						
$Ru(CO)(H)_2(PMe_3)_3 + OH^- \rightarrow [Ru(CO_2H)(H)_2(PMe_3)_3]^-$	-126.6	159.5	49.8	3.8	82.7	36.7
$Ru(CO)(H)_2(PF_3)_3 + OH^- \rightarrow [Ru(CO_2H)(H)_2(PF_3)_3]^-$	-304.1	188.0	54.9	9.1	-61.2	-107.0
$[Ru(CO)_3Cl_3]^- + OH^- \rightarrow [Ru(CO)_2(CO_2H)Cl_2]^- + Cl^-$	-208.7	63.8	20.0	12.7	-124.8	-132.1
$[Ru(CO)_3Cl_3]^- + OH^- \rightarrow [Ru(CO)_2(CO_2H)Cl_3]^{2-}$	-41.2	-131.3	53.6	13.7	-119.0	-158.8

[a] B97-D/ECP2 energies; [b] model solvent MeOH and [c] RI-BP86/ECP1 energies.

Table S2. Reaction Energies in gas phase (***ΔE$_{GAS}$***)[a]; Solvation (***δE$_{solv}$***)[a,b] and Thermochemistry (***δE$_G$*** and ***δE$_H$***)[c]; Final Free Energies (***ΔG***) in kJ/mol and Enthalpies (***ΔH***) for the OH^- uptake to the carbonyl complexes of iron.

Reaction	ΔE_{GAS}	δE_{solv}	δE_G	δE_H	ΔG	ΔH
$FeCO_5 + OH^- \rightarrow [Fe(CO)_4(CO_2H)]^-$	-312.4	173.2	51.8	12.8	-87.4	-126.3
PMe$_3$ Ligands						
$Fe(CO)_4(PMe_3) + OH^- \rightarrow [Fe(CO)_3(CO_2H)(PMe_3)]^-$	-233.1	157.2	48.2	9.9	-27.7	-66.0
$Fe(CO)_3(PMe_3)_2 + OH^- \rightarrow [Fe(CO)_2(CO_2H)(PMe_3)_2]^-$	-162.9	168.3	51.7	8.1	57.1	13.5
$Fe(CO)_2(PMe_3)_3 + OH^- \rightarrow [Fe(CO)(CO_2H)(PMe_3)_3]^-$	-133.7	192.1	48.5	5.7	106.9	64.1
$Fe(CO)(PMe_3)_4 + OH^- \rightarrow [Fe(CO_2H)(PMe_3)_4]^-$	-92.7	199.5	46.1	2.9	152.9	109.7
PF$_3$ Ligands						
$Fe(CO)_4(PF_3) + OH^- \rightarrow [Fe(CO)_3(CO_2H)(PF_3)]^-$	-321.0	177.5	53.8	11.6	-89.7	-132.0
$Fe(CO)_3(PF_3)_2 + OH^- \rightarrow [Fe(CO)_2(CO_2H)(PF_3)_2]^-$	-354.7	188.7	55.5	11.8	-110.5	-154.3
$Fe(CO)_2(PF_3)_3 + OH^- \rightarrow [Fe(CO)(CO_2H)(PF_3)_3]^-$	-359.5	199.5	64.1	10.9	-95.9	-149.1
$Fe(CO)(PF_3)_4 + OH^- \rightarrow [Fe(CO_2H)(PF_3)_4]^-$	-380.1	199.4	58.0	11.2	-122.7	-169.5
py and bipy Ligands						
$Fe(CO)_4(py) + OH^- \rightarrow [Fe(CO)_3(CO_2H)(py)]^-$	-235.2	150.9	52.9	10.5	-31.4	-73.9
$Fe(CO)_3(bipy) + OH^- \rightarrow Fe(CO)_2(CO_2H)(bipy)]^-$	-226.3	178.0	51.2	8.6	2.8	-39.7
Octahedral Complexes						
$Fe(CO)(H)_2(PMe_3)_3 + OH^- \rightarrow [Fe(CO_2H)(H)_2(PMe_3)_3]^-$	-108.4	164.6	54.6	5.6	110.8	61.8
$Fe(CO)(H)_2(PF_3)_3 + OH^- \rightarrow [Fe(CO_2H)(H)_2(PF_3)_3]^-$	-315.8	189.1	58.2	8.8	-68.5	-117.9

[a] B97-D/ECP2 energies; [b] model solvent MeOH and [c] RI-BP86/ECP1 energies.

Table S3. Reaction Energies in gas phase (*ΔE_{GAS}*)[a]; Solvation (*δE_{solv}*)[a,b] and Thermochemistry (*δE_G* and *δE_H*)[c]; Final Free Energies (*ΔG*) in kJ/mol and Enthalpies (*ΔH*) for the OH^- uptake to the carbonyl complexes of osmium.

Reaction	ΔE_{GAS}	δE_{solv}	δE_G	δE_H	ΔG	ΔH
$OsCO_5 + OH^- \rightarrow [Os(CO)_4(CO_2H)]^-$	-326.8	175.2	51.8	12.9	-99.8	-138.7
PMe_3 Ligands						
$Os(CO)_4(PMe_3) + OH^- \rightarrow [Os(CO)_3(CO_2H)(PMe_3)]^-$	-239.4	152.5	45.2	6.7	-41.7	-80.2
$Os(CO)_3(PMe_3)_2 + OH^- \rightarrow [Os(CO)_2(CO_2H)(PMe_3)_2]^-$	-169.8	161.7	51.1	5.8	43.0	-2.3
$Os(CO)_2(PMe_3)_3 + OH^- \rightarrow [Os(CO)(CO_2H)(PMe_3)_3]^-$	-153.9	194.1	47.9	3.0	88.1	43.2
$Os(CO)(PMe_3)_4 + OH^- \rightarrow [Os(CO_2H)(PMe_3)_4]^-$	-121.0	192.4	47.1	1.4	118.5	72.9
PF_3 Ligands						
$Os(CO)_4(PF_3) + OH^- \rightarrow [Os(CO)_3(CO_2H)(PF_3)]^-$	-338.7	180.3	53.8	11.9	-104.7	-146.6
$Os(CO)_3(PF_3)_2 + OH^- \rightarrow [Os(CO)_2(CO_2H)(PF_3)_2]^-$	-377.7	191.8	54.3	12.4	-131.6	-173.5
$Os(CO)_2(PF_3)_3 + OH^- \rightarrow [Os(CO)(CO_2H)(PF_3)_3]^-$	-372.2	201.5	55.9	11.3	-114.8	-159.3
$Os(CO)(PF_3)_4 + OH^- \rightarrow [Os(CO_2H)(PF_3)_4]^-$	-402.5	203.9	57.1	11.6	-141.6	-187.1
py and bipy Ligands						
$Os(CO)_4(py) + OH^- \rightarrow [Os(CO)_3(CO_2H)(py)]^-$	-245.3	147.0	51.8	11.0	-46.5	-87.3
$Os(CO)_3(bipy) + OH^- \rightarrow Os(CO)_2(CO_2H)(bipy)]^-$	-265.9	181.6	53.9	9.8	-30.4	-74.5
Octahedral Complexes						
$Os(CO)(H)_2(PMe_3)_3 + OH^- \rightarrow [Os(CO_2H)(H)_2(PMe_3)_3]^-$	-123.4	161.4	51.2	4.3	89.2	42.2
$Os(CO)(H)_2(PF_3)_3 + OH^- \rightarrow [Os(CO_2H)(H)_2(PF_3)_3]^-$	-304.8	187.4	53.6	9.7	-63.8	-107.6

[a] B97-D/ECP2 energies; [b] model solvent MeOH and [c] RI-BP86/ECP1 energies.

Table S4. Natural charge in units of elementary charge of the hydroxide fragment in the metalla-acid product of ruthenium carbonyl complexes (B97-D/ECP2/PCM level).

Complexes	Natural Charges		
	**O**	**H**	**OH^-**
$[Ru(CO)_4(CO_2H)]^-$	-0.722	0.496	-0.226
$[Ru(CO)_3(CO_2H)(PMe_3)]^-$	-0.741	0.496	-0.245
$[Ru(CO)_2(CO_2H)(PMe_3)_2]^-$	-0.779	0.497	-0.282
$[Ru(CO)(CO_2H)(PMe_3)_3]^-$	-0.794	0.490	-0.304
$[Ru(CO_2H)(PMe_3)_4]^-$	-0.816	0.496	-0.320
$[Ru(CO)_3(CO_2H)(PF_3)]^-$	-0.713	0.499	-0.214
$[Ru(CO)_2(CO_2H)(PF_3)_2]^-$	-0.712	0.495	-0.216
$[Ru(CO)(CO_2H)(PF_3)_3]^-$	-0.707	0.494	-0.213
$[Ru(CO_2H)(PF_3)_4]^-$	-0.698	0.497	-0.201
$[Ru(CO)_3(CO_2H)(py)]^-$	-0.742	0.495	-0.247
$[Ru(CO)_2(CO_2H)(bipy)]^-$	-0.726	0.497	-0.229
$[Ru(CO_2H)(H)_2(PMe_3)_3]^-$	-0.812	0.503	-0.309
$[Ru(CO_2H)(H)_2(PF_3)_3]^-$	-0.735	0.490	-0.245
$[Ru(CO)_2(CO_2H)Cl_2]^-$	-0.696	0.504	-0.192
$[Ru(CO)_2(CO_2H)Cl_3]^{2-}$	-0.730	0.493	-0.237

Table S5. Relative free Energies (and free enthalpies in parenthesis) in kJ/mol (B97-D) with that of the lowest stereoisomer set to 0.0 in each case (P=PMe$_3$, py= pyridine).

$[Fe(CO)_3(CO_2H)(PMe_3)]^-$		$Fe(CO)_4(PMe_3)$	
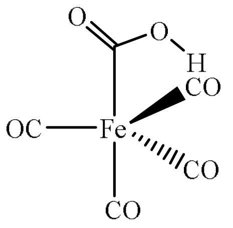	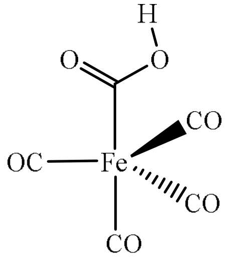	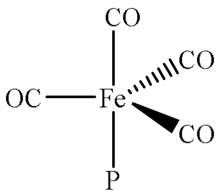	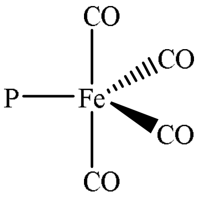
**0.0 (0.0)**	**3.1 (5.9)**	**0.0 (0.0)**	**20.0 (23.4)**
$[Fe(CO)_3(CO_2H)(PMe_3)]^-$		$[Fe(CO)_3(CO_2H)(PMe_3)]^-$	
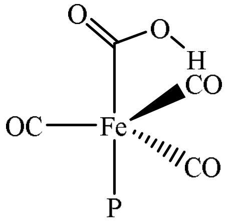	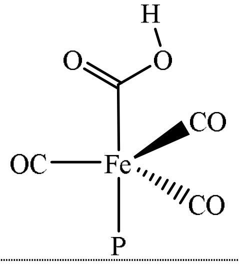	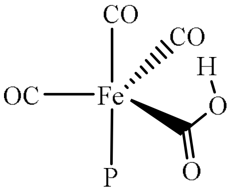	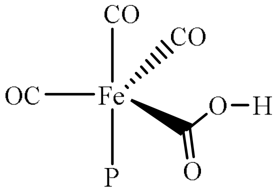
**0.0 (0.0)**	**4.2 (0.9)**	**0.0 (0.0)**	**2.5 (5.1)**
$[Fe(CO)_3(CO_2H)(PMe_3)]^-$		$Fe(CO)_3(PMe_3)_2$	
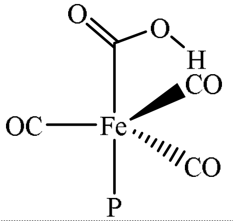	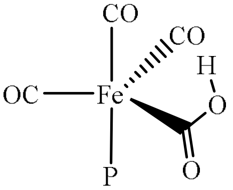	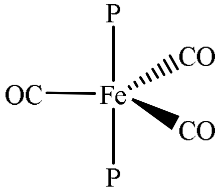	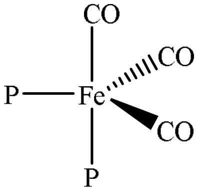
**0.0 (0.0)**	**30.9 (23.3)**	**0.0 (0.0)**	**40.4 (35.5)**
$[Fe(CO)_2(CO_2H)(PMe_3)_2]^-$		$[Fe(CO)(CO_2H)(PMe_3)_3]^-$	
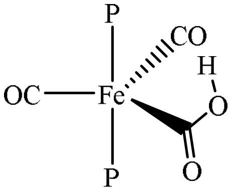	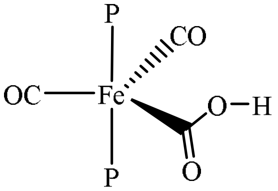	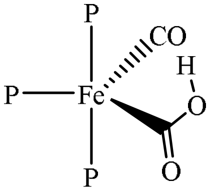	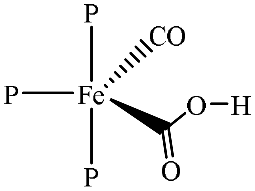
**0.0 (0.0)**	**7.0 (12.0)**	**0.0 (0.0)**	**12.8 (13.7)**
$[Fe(CO_2H)(PMe_3)_4]^-$		$[Fe(CO_2H)(H)_2(PMe_3)_3]^-$	
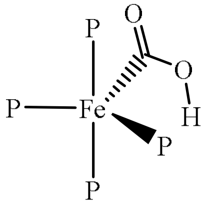	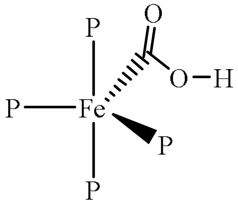	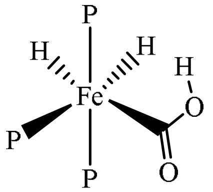	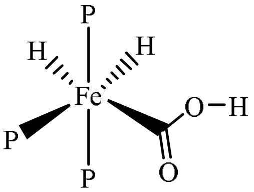
0.0 (0.0)	18.6 (20.6)	0.0 (0.0)	18.1 (23.2)
$[Fe(CO)_3(CO_2H)(py)]^-$		$[Fe(CO)_3(CO_2H)(py)]^-$	

**0.0 (0.0)**	**4.6 (9.8)**	**0.0 (0.0)**	**6.0 (8.1)**
**0.0 (0.0)**	**21.8 (21.1)**	**0.0 (0.0)**	**11.9 (11.6)**

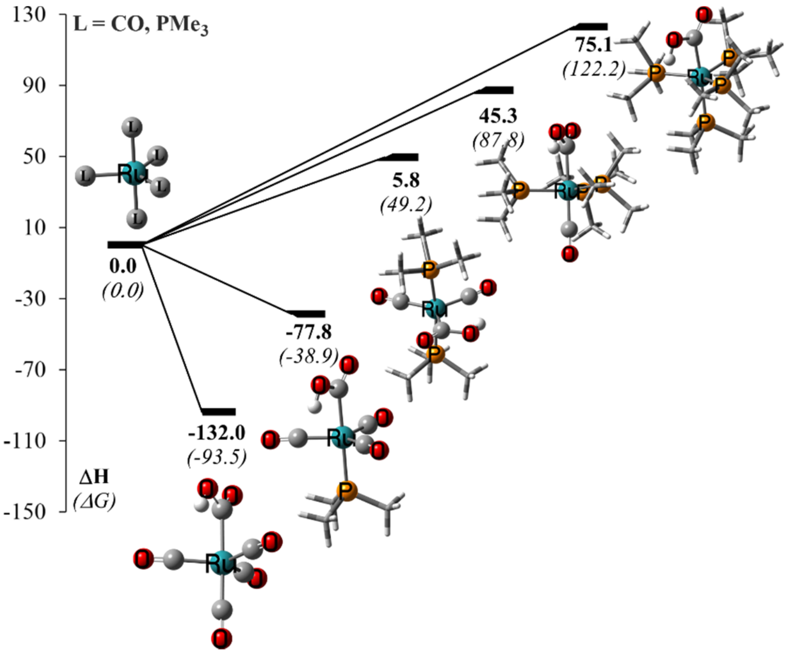

Figure S1. Computed free energies (B97-D level, kJ/mol) for the OH^- uptake of the carbonyl reactant. The number of PMe_3 ligands increases from left to right.

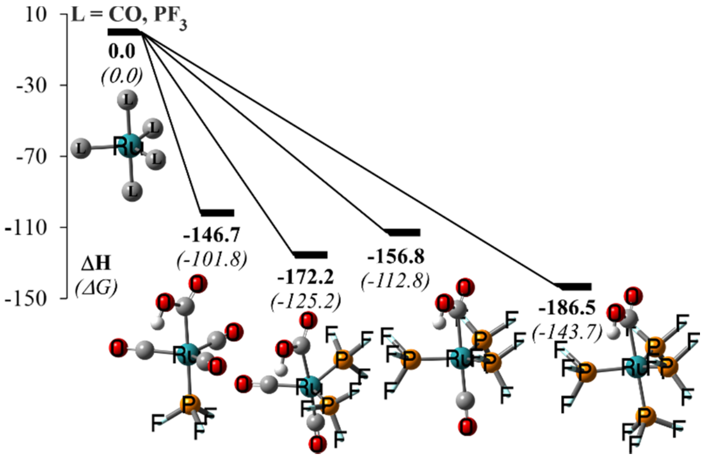

Figure S2. Relative free energy (kJ/mol) for the OH^- uptake with that of the respective carbonyl reactant set to 0.0 kJ/mol in each case. The number of PF_3 ligands increases from left to right.

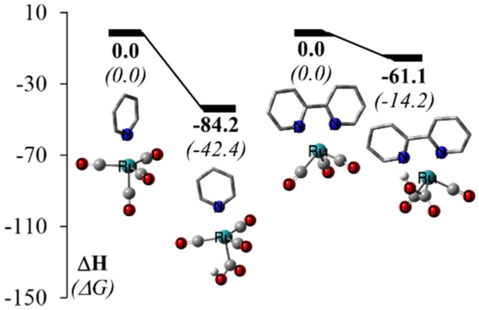

Figure S3. Relative free energy (kJ/mol) for the OH^- uptake on replacing one CO ligand with py (left) and two CO ligands with bipy (right).

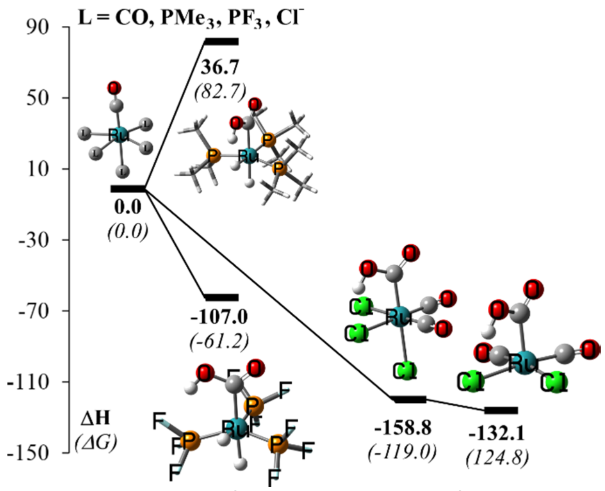

Figure S4. Relative free energy (kJ/mol) for the OH^- uptake with that of the respective octahedral carbonyl reactant set to 0.0 kJ/mol in each case.

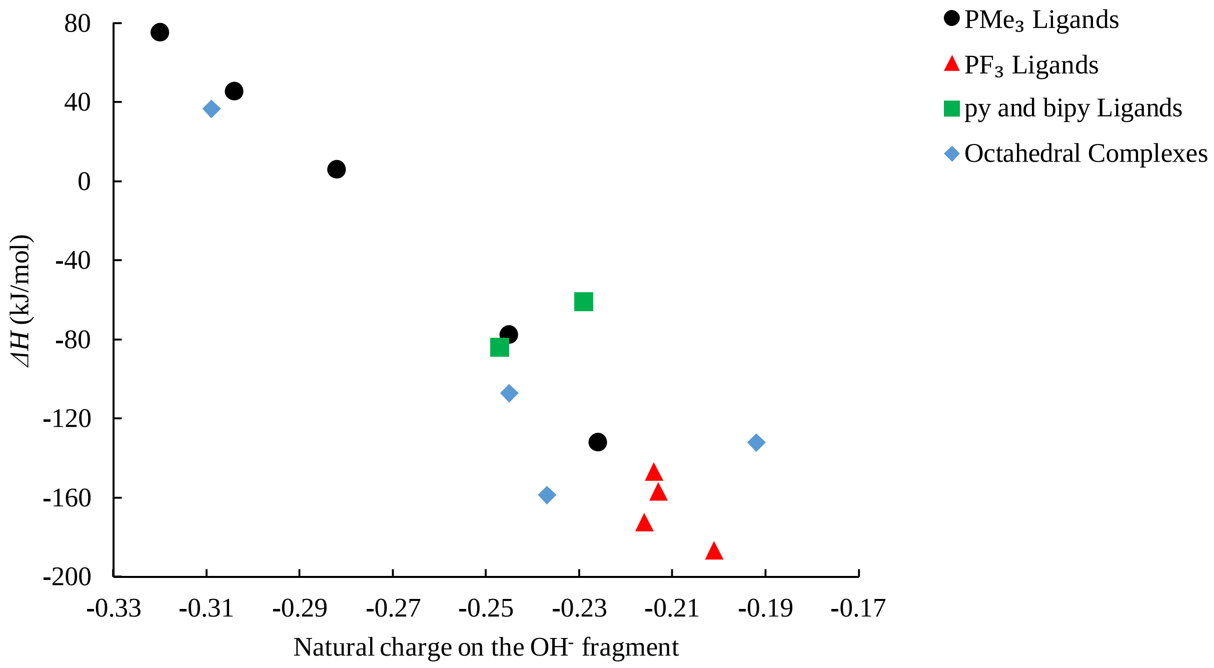

Figure S5. Plot of computed (B97-D level) enthalpies of OH^- uptake vs. natural charge on the OH^- fragment of the metalla-acids.

Atomic coordinates (in Å) of ruthenium complexes, optimised at the RI-BP86/ECP1 level, and corresponding SCF energies (in a.u.).

$RuCO_5$
SCF done: -661.812026

Ru	0.000000	0.000000	0.000000
C	0.000000	0.000000	1.952856
C	0.000000	1.949720	0.000000
C	1.688508	-0.974860	0.000000
C	-1.688507	-0.974861	0.000000
C	0.000000	0.000000	-1.952856
O	0.000000	0.000000	3.113937
O	2.698368	-1.557903	0.000000
O	0.000000	3.115807	0.000000
O	0.000000	0.000000	-3.113937
O	-2.698367	-1.557904	0.000000

$[Ru(CO)_4(CO_2H)]^-$
SCF done: -737.728247

Ru	0.303180	0.000078	-0.014912
C	2.218656	0.001244	-0.233655
C	0.083303	1.730995	-0.834966
C	0.373739	-0.008549	1.911072
C	0.083419	-1.723456	-0.850332
O	-0.004453	2.808269	-1.312656
O	-0.004356	-2.796501	-1.337439
O	3.386523	0.001736	-0.379036
O	0.433902	-0.013865	3.085964
C	-1.873650	-0.000985	0.165308
O	-2.580552	-0.005898	1.158648
O	-2.513718	0.004994	-1.079038
H	-1.767877	0.008106	-1.717051

$Ru(CO)_4(PMe_3)$
SCF done: -1009.624647

Ru	-0.768152	-0.000829	0.000574
C	-2.692712	0.002499	0.001640
C	-0.697699	1.280175	1.445712
C	-0.706355	-1.892458	0.389898
C	-0.700061	0.608103	-1.832460
O	-3.859090	0.004549	0.002919
O	-0.667853	-3.041575	0.627526
O	-0.652622	2.058034	2.323996
O	-0.657262	0.978099	-2.945884
P	1.630806	-0.001228	-0.000919
C	2.411476	-0.588087	1.567621
H	2.079668	0.054339	2.398247
H	3.512039	-0.563730	1.499048
H	2.078250	-1.616981	1.774500
C	2.406367	1.653048	-0.276043
H	2.069348	2.344286	0.512192

[Ru(CO)3(CO2H)(PMe3)]⁻
SCF done: -1085.512837

Ru	-0.399924	0.037452	-0.000319
C	-2.540968	0.109086	-0.003389
C	-0.488687	-0.804098	-1.718422
C	-0.355826	1.948804	0.015970
C	-0.489933	-0.830861	1.704279
O	-3.308127	1.059215	-0.001256
O	-0.304628	3.132283	0.024870
O	-0.484713	-1.302818	-2.798428
O	-0.483685	-1.344891	2.777118
P	1.955097	-0.083639	0.002507
C	2.852921	0.762111	-1.393618
H	2.508336	0.326650	-2.345486
H	3.951047	0.662307	-1.315169
H	2.576211	1.828578	-1.391804
C	2.724539	-1.779691	-0.069904
H	2.368267	-2.283622	-0.982965

H	2.073671	2.051229	-1.247327
H	3.507124	1.585397	-0.262571
C	2.407519	-1.065502	-1.296727
H	2.074489	-2.105892	-1.157346
H	3.508124	-1.019992	-1.243505
H	2.072823	-0.728234	-2.290203

H	2.368767	-2.361362	0.795903
H	3.829701	-1.749981	-0.068428
C	2.838452	0.636924	1.475674
H	2.561288	1.699732	1.563642
H	3.937230	0.543702	1.400897
H	2.482956	0.121105	2.382318
H	-2.326890	-1.759710	-0.009947
O	-3.115387	-1.173557	-0.008644

$Ru(CO)_3(PMe_3)_2$

SCF done: -1357.42703541

Ru	-0.000010	0.004758	0.005325
C	-0.001820	-0.615914	1.817817
C	-0.000422	-1.257904	-1.436290
C	0.002919	1.885759	-0.362345
O	-0.004567	-0.999598	2.936246
O	0.000167	-2.037868	-2.325038
O	0.006397	3.046157	-0.589223
P	-2.360957	-0.000962	-0.004348
C	-3.151157	-1.667607	0.136890
H	-2.816274	-2.300685	-0.699861
H	-4.252393	-1.597693	0.129776
H	-2.819075	-2.141147	1.074335
C	-3.150163	0.703108	-1.522620
H	-2.807644	0.134261	-2.401472
H	-2.825755	1.748548	-1.644894
H	-4.251364	0.664114	-1.462417
C	-3.172784	0.951365	1.360134
H	-2.837324	0.548979	2.328822
H	-4.272907	0.891969	1.294320
H	-2.859001	2.005469	1.299437
P	2.360569	-0.002995	-0.002424
C	3.149434	0.378018	-1.633306
H	2.809240	1.369064	-1.972506
H	2.820444	-0.368632	-2.373223
H	4.250719	0.367296	-1.564316
C	3.169188	1.209607	1.137686
H	2.854631	0.998842	2.172004
H	2.835161	2.226903	0.879215
H	4.269449	1.157324	1.070458
C	3.154334	-1.605529	0.475166
H	2.834349	-1.873211	1.494496
H	4.254999	-1.540685	0.436974
H	2.805854	-2.395640	-0.208523

$[Ru(CO)_2(CO_2H)(PMe_3)_2]^-$

SCF done: -1433.284752

Ru	0.001797	-0.315113	0.150636
C	-0.000203	-1.739287	-1.084210
C	0.008133	1.801369	-0.374166
C	0.004501	-0.719796	1.999428
O	0.000020	-2.686263	-1.821968
O	0.038955	2.401547	-1.449772
O	0.006009	-1.125158	3.128495
P	-2.292957	-0.088798	-0.083178
C	-2.869137	0.495240	-1.754331
H	-2.301441	1.407554	-1.997692
H	-3.957303	0.689957	-1.788483
H	-2.603224	-0.273897	-2.497532
C	-3.164027	1.115918	1.041563
H	-2.676792	2.097829	0.935742
H	-3.029114	0.779401	2.082678
H	-4.242528	1.199773	0.813449
C	-3.350587	-1.605794	0.153532
H	-3.002576	-2.383118	-0.545452
H	-4.423962	-1.403565	-0.017349
H	-3.201898	-1.981179	1.179030
P	2.295962	-0.091415	-0.086245
C	3.165265	1.126706	1.024889
H	3.024155	0.806347	2.070205
H	2.683206	2.109158	0.902861
H	4.245112	1.203397	0.800559
C	3.353667	-1.605087	0.170291
H	3.006816	-2.391284	-0.519291
H	3.203646	-1.967574	1.200252
H	4.427214	-1.404844	-0.001805
C	2.868525	0.474063	-1.764301
H	2.610179	-0.307579	-2.497077
H	3.954834	0.678322	-1.800375
H	2.291132	1.377647	-2.018278
O	-0.032200	2.641114	0.784322
H	-0.043798	1.953612	1.493197

$Ru(CO)_2(PMe_3)_3$

SCF done: -1705.201439

Ru	-0.003884	-0.309081	-0.022350
C	-0.010807	-1.089843	1.705094
C	0.002811	-1.045066	-1.773643
O	-0.015236	-1.648930	2.755500
O	0.007721	-1.572618	-2.839192
P	-2.333857	-0.607540	-0.006420
C	-3.315043	0.205152	1.348441
H	-2.906256	-0.115947	2.319742
H	-4.383825	-0.064589	1.293593
H	-3.215714	1.300051	1.280328
C	-2.856202	-2.377143	0.204036
H	-2.439186	-2.762507	1.147638
H	-2.434329	-2.971343	-0.621662
H	-3.955463	-2.482330	0.213053
C	-3.294295	-0.139081	-1.525820
H	-3.188961	0.939339	-1.717062
H	-4.363640	-0.390561	-1.419964
H	-2.873066	-0.681572	-2.386874
P	-0.007360	2.070440	0.005005
C	1.414468	3.016140	-0.761177
H	1.500057	2.743938	-1.825842
H	2.359162	2.751189	-0.259833
H	1.267832	4.107924	-0.678490
C	-1.395249	3.009819	-0.829130
H	-2.360862	2.746961	-0.367944
H	-1.434334	2.731362	-1.894836
H	-1.253599	4.102248	-0.745825
C	-0.051953	2.904398	1.678268

$[Ru(CO)(CO_2H)(PMe_3)_3]^-$

SCF done: -1781.055799

Ru	0.000106	-0.059585	-0.219316
C	0.000387	-0.157186	-2.099973
C	-0.000222	0.025291	1.932703
O	0.000561	-0.235322	-3.293800
O	-0.000225	0.956600	2.745849
P	2.095708	-1.035055	-0.113605
C	3.335704	-0.488167	-1.415362
H	2.894582	-0.644793	-2.413361
H	4.297756	-1.032070	-1.348492
H	3.516079	0.592553	-1.293793
C	2.336787	-2.896601	-0.344185
H	1.848366	-3.194697	-1.286943
H	1.826310	-3.423864	0.479505
H	3.402341	-3.203603	-0.367615
C	3.170429	-0.822056	1.406267
H	3.355078	0.253664	1.558596
H	4.135809	-1.354826	1.313760
H	2.615693	-1.193798	2.282000
P	0.000404	2.254984	-0.131662
C	-1.409574	3.118493	0.750960
H	-1.430459	2.699482	1.769828
H	-2.356400	2.875458	0.240528
H	-1.284749	4.218277	0.797387
C	1.410337	3.118117	0.751399
H	2.357257	2.874831	0.241261
H	1.430794	2.699100	1.770273
H	1.285790	4.217934	0.797788
C	0.000783	3.225725	-1.732126

H	-0.953675	2.573365	2.218393
H	-0.052228	4.007543	1.606529
H	0.820820	2.575785	2.264957
P	2.325418	-0.612904	0.009762
C	2.837513	-2.388463	0.193829
H	2.418790	-2.966075	-0.645164
H	2.411508	-2.787950	1.127464
H	3.936162	-2.499385	0.209106
C	3.299734	0.170221	1.387072
H	3.211373	1.267090	1.337476
H	4.366533	-0.108179	1.336655
H	2.880035	-0.163812	2.349274
C	3.302908	-0.124278	-1.492562
H	3.209986	0.958660	-1.663891
H	2.884126	-0.646514	-2.367254
H	4.368868	-0.388124	-1.382213

H	0.890680	2.949976	-2.321289
H	0.000902	4.318373	-1.557734
H	-0.889006	2.950215	-2.321565
P	-2.095788	-1.034496	-0.114266
C	-2.337287	-2.895976	-0.344939
H	-1.827208	-3.423382	0.478906
H	-1.848650	-3.194191	-1.287547
H	-3.402915	-3.202697	-0.368708
C	-3.335233	-0.487266	-1.416404
H	-3.515358	0.593500	-1.294880
H	-4.297451	-1.030914	-1.349840
H	-2.893842	-0.643999	-2.414268
C	-3.170925	-0.821226	1.405273
H	-3.355338	0.254542	1.557552
H	-2.616561	-1.193121	2.281176
H	-4.136418	-1.353739	1.312461
H	-0.000434	-1.839354	1.732692
O	-0.000493	-1.274342	2.542828

$Ru(CO)(PMe_3)4$

SCF done: -2052.960469

Ru	-0.028237	-0.013229	-0.307435
C	-0.094569	-0.290471	-2.153908
O	-0.146233	-0.493691	-3.332422
P	-2.368472	0.061585	-0.584716
C	-3.112758	-1.489308	-1.306502
H	-2.571294	-1.751483	-2.228399
H	-4.185554	-1.356158	-1.532236
H	-2.996659	-2.320395	-0.592181
C	-2.966768	1.302490	-1.840649
H	-2.443134	1.115889	-2.790817
H	-2.709819	2.320425	-1.507012
H	-4.058217	1.233409	-1.995865
C	-3.619871	0.379952	0.768966
H	-3.474461	-0.319269	1.605540
H	-4.642770	0.258263	0.372033
H	-3.511154	1.405003	1.150661
P	-0.042990	-1.809617	1.215007
C	1.373540	-2.074728	2.418786
H	1.478169	-1.181226	3.055751
H	2.317703	-2.214341	1.867076
H	1.209586	-2.960859	3.058665
C	-1.439153	-2.031845	2.448702
H	-2.385617	-2.197105	1.908445
H	-1.542680	-1.116077	3.052980
H	-1.265105	-2.894272	3.117360
C	-0.089931	-3.533811	0.474207
H	-0.994038	-3.627683	-0.149069
H	-0.091029	-4.328974	1.242883
H	0.778553	-3.669105	-0.189994
P	2.286617	-0.122767	-0.691530
C	2.905469	0.888998	-2.129107
H	2.678939	1.952286	-1.957289
H	2.370463	0.573238	-3.037701
H	3.992719	0.762025	-2.275194
C	2.953394	-1.790238	-1.199237
H	2.843459	-2.510464	-0.372688
H	4.018661	-1.731853	-1.484223
H	2.364061	-2.157042	-2.054274
C	3.570013	0.353363	0.582648
H	3.421408	-0.217299	1.511747
H	3.481853	1.425343	0.819579
H	4.587201	0.161189	0.199009
P	0.145151	2.006521	0.913847
C	-1.358081	3.027388	1.381793
H	-1.979221	3.234576	0.495483
H	-1.052536	3.988309	1.832517
H	-1.965284	2.475764	2.116794
C	1.135425	3.408855	0.149295
H	0.677562	3.682696	-0.814949
H	2.167182	3.079040	-0.051924
H	1.171181	4.298368	0.804816
C	0.934017	2.025433	2.622502
H	0.328361	1.391650	3.292237
H	0.999944	3.043840	3.049065
H	1.944911	1.591567	2.573578

$[Ru(CO_2H)(PMe_3)_4]^-$

SCF done: -2128.801482

Ru	0.034148	0.155290	0.094172
C	0.300346	2.239148	0.315880
O	0.376946	3.198569	-0.459655
P	-2.003758	0.452557	1.155975
C	-2.590602	2.178645	1.617527
H	-1.879695	2.641787	2.316668
H	-3.605077	2.152153	2.057910
H	-2.601256	2.798828	0.706511
C	-2.471726	-0.390502	2.789744
H	-1.790606	-0.016135	3.572355
H	-2.314798	-1.479630	2.706512
H	-3.522002	-0.200929	3.092872
C	-3.592232	-0.005324	0.224504
H	-3.646844	0.626520	-0.678130
H	-4.504664	0.155516	0.832502
H	-3.558618	-1.055787	-0.102021
P	-0.330145	0.384475	-2.165818
C	1.126768	0.376451	-3.371298
H	1.680242	-0.570679	-3.253141
H	1.804267	1.202406	-3.097171
H	0.822381	0.493679	-4.431648
C	-1.407989	-0.829476	-3.136216
H	-2.424654	-0.831166	-2.706675
H	-1.005535	-1.853279	-3.053765
H	-1.471863	-0.560759	-4.209221
C	-1.153013	1.946036	-2.796451
H	-2.195668	1.964013	-2.435056
H	-1.147904	2.011189	-3.901468
H	-0.618272	2.793878	-2.338778
P	2.291990	0.160199	0.655534
C	2.906978	0.267104	2.450242
H	2.497987	-0.586337	3.017087
H	2.506817	1.195246	2.889720
H	4.013568	0.273471	2.534133
C	3.335721	1.574476	-0.012238
H	3.294806	1.550919	-1.113365
H	4.387856	1.501177	0.322305
H	2.903585	2.530008	0.319187
C	3.483671	-1.212690	0.142270
H	3.454242	-1.322016	-0.954284
H	3.194702	-2.174269	0.598031
H	4.519629	-0.977783	0.452336
P	-0.117381	-2.173865	0.134902
C	-1.760371	-3.069790	-0.113748
H	-2.454777	-2.796765	0.696796
H	-1.631656	-4.168457	-0.113297
H	-2.208599	-2.757362	-1.070860
C	0.370097	-3.095042	1.707331
H	-0.305586	-2.782348	2.520704
H	1.390952	-2.798201	1.996748
H	0.323425	-4.196082	1.594135
C	0.857737	-3.247475	-1.078678
H	0.572184	-2.975102	-2.108291
H	0.665700	-4.326470	-0.924466
H	1.935086	-3.055652	-0.970528
O	0.497479	2.592210	1.690677
H	0.379805	1.695594	2.101783

$Ru(CO)_4(PF_3)$

SCF done: -1189.467548

Ru	0.623253	0.001036	-0.000648
C	2.555987	-0.000928	-0.005066
C	0.639194	1.884153	-0.494070
C	0.634146	-1.370242	-1.382524
C	0.641014	-0.509613	1.877682
O	3.718152	-0.001640	-0.007838
O	0.674498	-2.191792	-2.209553
O	0.683223	3.011713	-0.789386
O	0.686280	-0.815707	3.002308
P	-1.656930	-0.000463	0.001922
F	-2.429361	1.342014	-0.384139
F	-2.425924	-0.338804	1.359213
F	-2.424705	-1.009081	-0.968523

$[Ru(CO)_3(CO_2H)(PF_3)]^-$

SCF done: -1265.388713

Ru	-0.312871	0.021380	0.000584
C	-2.467862	0.091418	-0.000278
C	-0.501477	-0.818975	-1.721838
C	-0.351598	1.948187	0.000673
C	-0.502156	-0.819359	1.722915
O	-3.214481	1.054005	-0.000390
O	-0.384227	3.123333	0.000350
O	-0.597292	-1.310114	-2.791572
O	-0.598227	-1.309889	2.792900
P	1.921135	-0.116859	0.000019
H	-2.269490	-1.782584	-0.000847
O	-3.044087	-1.179174	-0.000705
F	2.860205	1.212875	-0.001103
F	2.713656	-0.865968	1.206475
F	2.712587	-0.866774	-1.206548

$Ru(CO)_3(PF_3)_2$

SCF done: -1717.122566

Ru	0.000081	0.000297	0.001374
C	-0.000236	-1.226772	1.508221
C	0.000525	1.919337	0.307460
C	-0.000148	-0.694602	-1.813396
O	-0.000395	-1.965521	2.411661
O	0.000812	3.071689	0.492062
O	-0.000232	-1.111282	-2.903490
P	2.258247	-0.000022	0.000426
P	-2.258076	0.001160	0.000515
F	3.026905	-0.349474	-1.354325
F	3.029505	-0.998099	0.978768
F	3.028866	1.347088	0.374092
F	-3.029804	-0.996403	0.979009
F	-3.026931	-0.348056	-1.354177
F	-3.028035	1.348695	0.374029

$[Ru(CO)_2(CO_2H)(PF_3)_2]^-$

SCF done: -1793.052404

Ru	-0.009424	-0.051433	0.587136
C	-0.110866	-1.795620	1.394125
C	0.046534	1.995210	-0.169560
C	-0.023148	0.829609	2.284284
O	-0.162016	-2.840583	1.928953
O	-0.864064	2.604870	-0.711697
O	-0.035833	1.343263	3.346033
P	-1.900761	-0.277959	-0.591273
P	1.812293	-0.469723	-0.589308
O	1.236444	2.691491	0.036712
H	1.851239	2.051405	0.451470
F	1.915923	-0.291943	-2.207821
F	3.192920	0.386041	-0.299368
F	2.524720	-1.932527	-0.538457
F	-2.565850	-1.759270	-0.733973
F	-3.279268	0.473035	-0.167700
F	-1.993082	0.110061	-2.163073

$Ru(CO)_2(PF3)_3$

SCF done: -2244.788192

Ru	-0.013497	-0.023633	-0.002523
C	-0.090186	-0.030924	1.942583
C	0.047047	-0.024611	-1.948205
O	-0.136830	-0.032978	3.103962
O	0.082293	-0.022914	-3.109989
P	-1.978323	-1.125800	-0.073557
P	0.018721	2.219107	0.002240
P	1.967476	-1.085132	0.065529
F	2.928837	-0.826645	1.325110
F	3.015870	-0.821023	-1.121374
F	2.063663	-2.690266	0.065178
F	0.775001	2.975878	-1.194767
F	0.687438	2.971879	1.252700
F	-1.342644	3.074492	-0.045124
F	-3.031217	-0.887274	1.114754
F	-2.944711	-0.883879	-1.332551
F	-2.039057	-2.733157	-0.077907

$[Ru(CO)(CO_2H)(PF_3)_3]^-$

SCF done: -2320.714555

Ru	0.010155	0.000037	0.248777
C	0.129334	0.000130	2.167725
C	-0.081025	-0.000163	-1.935825
O	0.181511	0.000177	3.340663
O	0.874486	-0.000338	-2.699892
P	-1.061010	1.923309	0.128975
P	2.229222	0.000447	-0.043293
P	-1.060309	-1.923648	0.129105
H	-1.993510	0.000081	-1.784342
O	-1.347408	-0.000144	-2.520177
F	-1.376434	2.781040	1.475320
F	-0.521876	3.180651	-0.754385
F	-2.586509	2.004693	-0.472650
F	-2.585904	-2.005662	-0.472132
F	-0.520831	-3.180704	-0.754441
F	-1.375038	-2.781562	1.475500
F	3.000536	-1.200490	-0.813333
F	3.187099	0.000949	1.274151
F	2.999971	1.201402	-0.813865

$Ru(CO)(PF_3)_4$

SCF done: -2772.437200

Ru	0.000014	-0.015260	0.372069
C	0.000087	-0.228854	2.289375
O	0.000215	-0.374676	3.448706
P	-2.254027	0.031907	0.514312
P	0.000112	1.895122	-0.828542
P	2.254079	0.031529	0.514086
P	-0.000230	-1.844135	-0.932935
F	-3.139700	-0.452468	-0.722020
F	-2.947569	-0.817954	1.670503
F	-2.973961	1.433227	0.780561

$[Ru(CO_2H)(PF_3)_4]^-$

SCF done: -2848.372954

Ru	-0.087661	0.045539	-0.002187
C	-1.885915	1.248544	-0.076463
O	-1.931587	2.465790	-0.181475
P	-0.849065	-0.830240	-1.875441
P	0.968559	2.014105	-0.119689
P	-0.827837	-0.601411	1.968320
P	1.786843	-1.176512	0.057968
O	-3.102051	0.578758	-0.002662
H	-2.899365	-0.375718	0.083605
F	-1.245227	0.461149	3.129178

F	-1.221937	2.174995	-1.838580
F	0.000214	3.336326	-0.114226
F	1.222166	2.174835	-1.838618
F	2.974257	1.432705	0.780431
F	2.947601	-0.818573	1.670111
F	3.139551	-0.452838	-0.722392
F	-0.000518	-1.763272	-2.541517
F	1.218640	-2.886518	-0.789486
F	-1.219103	-2.886459	-0.789061

F	0.044425	-1.579622	2.934544
F	-2.223031	-1.460503	2.095776
F	0.755639	3.163823	1.002934
F	0.798697	3.001730	-1.394099
F	2.598999	2.058383	-0.094694
F	1.705133	-2.794026	0.134972
F	2.863147	-1.005807	1.256847
F	2.862114	-1.121325	-1.152787
F	0.028948	-1.895052	-2.740017
F	-2.227698	-1.721109	-1.885840
F	-1.299468	0.091614	-3.138817

$Ru(CO)_4(py)$

SCF done: -796.768570

Ru	-0.974139	-0.013656	-0.000014
C	-0.878319	-0.922806	-1.715822
C	-1.111351	1.919856	0.004365
C	-0.879113	-0.930418	1.711769
C	-2.854705	-0.157771	-0.000670
O	-0.840840	-1.470835	2.751580
O	-1.262899	3.083832	0.006991
O	-4.019022	-0.254870	-0.001107
O	-0.839473	-1.458607	-2.757991
C	1.947805	1.204779	0.000154
C	1.925655	-1.123442	0.000271
C	3.346150	1.229305	0.000065
H	1.367097	2.128849	0.000093
C	3.323098	-1.176601	0.000210
H	1.316713	-2.030206	0.000247
C	4.053967	0.019382	0.000108
H	3.861601	2.193567	-0.000044
H	3.819911	-2.150567	0.000214
H	5.148118	0.009135	0.000042
N	1.233652	0.048199	0.000302

$[Ru(CO)_3(CO_2H)(py)]^-$

SCF done: -872.655026

Ru	0.740577	-0.001487	0.019101
C	0.710759	-0.856570	1.734666
C	0.877149	1.906974	-0.006375
C	0.757286	-1.171635	-1.500028
C	2.831912	-0.078231	0.041859
O	0.717535	-1.914181	-2.427928
O	0.919853	3.097783	0.002983
O	3.598230	-0.520657	0.885842
O	0.670761	-1.384414	2.793675
C	-2.226040	1.212438	-0.072887
C	-2.216538	-1.105254	0.008665
C	-3.624405	1.246687	-0.103142
H	-1.632943	2.130702	-0.092154
C	-3.614176	-1.155174	-0.022436
H	-1.607437	-2.012160	0.058334
C	-4.344909	0.041815	-0.079354
H	-4.134003	2.214832	-0.145728
H	-4.115240	-2.128372	0.000446
H	-5.439748	0.036351	-0.103568
N	-1.507853	0.057744	-0.018249
H	2.631141	0.757041	-1.636079
O	3.413864	0.471498	-1.115561

$Ru(CO)_3(bipy)$

SCF done: -930.540360

Ru	-1.063177	0.037267	-0.023574
C	1.853189	-0.692936	-0.011122
C	0.503232	-2.631339	-0.115035
C	3.012413	-1.505384	-0.019128
C	1.612732	-3.465429	-0.126428
H	-0.513833	-3.028582	-0.147463
C	2.902617	-2.889905	-0.078115
H	3.997927	-1.034890	0.020874
H	1.470764	-4.548217	-0.172391
H	3.797959	-3.518106	-0.085707
C	1.867117	0.757557	0.053688
C	0.568231	2.723651	0.108138
C	3.047888	1.532992	0.126982
C	1.698373	3.524582	0.176826
H	-0.432072	3.161956	0.099707
C	2.976931	2.920024	0.187211
H	4.020454	1.033822	0.137238
H	1.581791	4.611001	0.220822
H	3.886397	3.524630	0.242314
N	0.595818	-1.268250	-0.062960
N	0.613223	1.353700	0.043552
C	-2.342075	1.419295	-0.084044
C	-1.788140	-0.708628	1.574531
C	-1.925980	-0.855346	-1.461728
O	-2.500599	-1.476336	-2.279197
O	-3.094298	2.319773	-0.121140
O	-2.326724	-1.237559	2.475254

$Ru(CO)_2(CO_2H)(bipy)]^-$

SCF done: -1006.429195

Ru	-1.012727	0.494449	-0.460646
C	1.333447	-1.343547	-0.108341
C	-0.665276	-2.586071	-0.499238
C	2.043345	-2.576154	0.015505
C	-0.004035	-3.791965	-0.392625
H	-1.740930	-2.548870	-0.691300
C	1.404152	-3.791794	-0.127762
H	3.116689	-2.542256	0.230307
H	-0.564446	-4.724590	-0.507999
H	1.958936	-4.731311	-0.033368
C	1.906001	-0.052693	0.028707
C	1.497777	2.304016	-0.001137
C	3.281031	0.218892	0.296950
C	2.823127	2.592080	0.244190
H	0.759206	3.102046	-0.118522
C	3.755051	1.512005	0.401967
H	3.965259	-0.627197	0.421642
H	3.141600	3.636553	0.315402
H	4.812906	1.707250	0.606222
N	-0.055511	-1.355135	-0.387099
N	0.988444	1.024342	-0.126912
C	-1.596535	2.243674	-0.788950
C	-1.745721	0.467677	1.487077
C	-2.592188	-0.170624	-1.203807
O	-3.566330	-0.621324	-1.703337
O	-1.928989	3.361734	-0.999371
O	-2.884292	0.337050	1.906282
H	0.098162	0.723648	1.916077
O	-0.724620	0.611744	2.439858

$Ru(CO)(H)_2(PMe_3)_3$

SCF done: -1593.049535

Ru	-0.069978	-0.412957	-0.023738
H	-0.211355	-2.007312	-0.382042
H	-0.067198	-0.267766	-1.691615
C	-0.084796	-0.689514	1.857079

$[Ru(CO_2H)(H)_2(PMe_3)_3]^-$

SCF done: -1668.891233

Ru	-0.022654	0.624287	-0.215836
P	-2.264089	0.978330	0.005959
P	2.213755	1.012505	-0.000793
P	-0.004522	-1.675794	0.007956

O	-0.092503	-0.876190	3.026035
P	-2.370888	-0.692951	-0.269519
P	2.159832	-1.023090	-0.279511
P	0.214779	1.933547	-0.050034
C	-3.634749	0.540314	0.338216
H	-3.555752	1.477406	-0.233274
H	-3.442555	0.763496	1.400255
H	-4.659581	0.143227	0.232420
C	-3.017345	-2.220592	0.571673
H	-2.461814	-3.093887	0.196812
H	-4.097830	-2.358620	0.391989
H	-2.833094	-2.142252	1.654987
C	-2.965276	-0.966249	-2.006885
H	-2.386273	-1.794396	-2.443811
H	-2.765800	-0.063412	-2.605285
H	-4.043007	-1.203741	-2.038568
C	-1.236695	3.085759	-0.285836
H	-0.908920	4.139242	-0.331747
H	-1.942231	2.968641	0.551338
H	-1.758753	2.832921	-1.222974
C	1.315453	2.611267	-1.395863
H	2.317292	2.157372	-1.328974
H	1.414625	3.709055	-1.331396
H	0.885361	2.336462	-2.372197
C	0.976525	2.738094	1.452383
H	1.957743	2.282205	1.658093
H	0.329513	2.545216	2.323503
H	1.101705	3.827710	1.323886
C	3.538745	-0.077214	0.552682
H	4.522357	-0.537534	0.353035
H	3.359844	-0.069686	1.640223
H	3.558031	0.966398	0.199870
C	2.798970	-1.128213	-2.019585
H	3.841674	-1.489242	-2.051834
H	2.734275	-0.136696	-2.493983
H	2.148047	-1.813414	-2.584421
C	2.553977	-2.730141	0.343417
H	3.615577	-2.988013	0.183362
H	1.910800	-3.454736	-0.179295
H	2.321322	-2.783907	1.418849

H	-0.035647	2.279254	-0.476889
H	-0.022282	0.953772	1.461385
C	-0.024504	0.359851	-2.322011
O	-0.036138	1.594554	-3.051301
H	-0.039493	2.222725	-2.290503
O	-0.017704	-0.648432	-3.049171
C	0.004687	-2.529298	1.682903
H	-0.885431	-2.209069	2.249317
H	0.013022	-3.633192	1.601968
H	0.891559	-2.195421	2.246521
C	1.398655	-2.599677	-0.820754
H	2.365112	-2.307845	-0.376745
H	1.287847	-3.698301	-0.752075
H	1.369466	-2.278712	-1.875871
C	-1.395680	-2.621458	-0.816389
H	-1.267627	-3.718203	-0.747793
H	-2.365194	-2.344486	-0.369547
H	-1.374647	-2.300356	-1.871656
C	-3.189010	0.159969	1.418462
H	-4.252564	0.461491	1.463462
H	-3.129204	-0.935820	1.311851
H	-2.679461	0.436118	2.355587
C	-3.464010	0.614347	-1.391572
H	-4.506205	0.899793	-1.152526
H	-3.122448	1.162583	-2.284515
H	-3.422044	-0.460323	-1.632092
C	-2.756630	2.748428	0.349743
H	-3.844618	2.862118	0.515785
H	-2.198368	3.089518	1.235633
H	-2.441517	3.367770	-0.504959
C	2.680330	2.789930	0.341364
H	2.119640	3.122554	1.228937
H	3.766959	2.920189	0.504096
H	2.353209	3.404302	-0.512410
C	3.415293	0.666542	-1.401554
H	3.063781	1.210424	-2.293303
H	4.453900	0.966672	-1.164892
H	3.388009	-0.408460	-1.642682
C	3.154846	0.208512	1.409280
H	3.111178	-0.888074	1.303048
H	4.213876	0.525969	1.451275
H	2.643777	0.477194	2.347745

$Ru(CO)(H)_2(PF_3)_3$

SCF done: -2132.635587

Ru	-0.000009	-0.347863	0.024963
H	-0.000011	-1.946964	-0.331568
H	-0.000093	-0.114157	-1.612119
C	0.000008	-0.797790	1.924299
O	0.000001	-1.101622	3.046438
P	-2.151547	-0.761860	-0.411029
P	2.151503	-0.761954	-0.411070
P	0.000042	1.914711	0.194693
F	3.220708	0.409400	-0.622892
F	2.981489	-1.665142	0.615836
F	2.452133	-1.587370	-1.741173
F	1.207759	2.733170	-0.468777
F	0.000069	2.629920	1.631031
F	-1.207634	2.733238	-0.468766
F	-2.981766	-1.664568	0.616110
F	-2.452184	-1.587695	-1.740870
F	-3.220547	0.409587	-0.623362

$[Ru(CO_2H)(H)_2(PF_3)_3]^-$

SCF done: -2208.539662

Ru	-0.001615	-0.310793	0.016420
H	0.002161	-1.784340	0.753322
H	-0.018061	-0.921960	-1.552281
C	0.159579	0.378500	2.060126
O	1.126615	0.922709	2.578545
P	-2.071380	-0.909324	-0.255944
P	2.085712	-0.967948	-0.202415
P	-0.015265	1.810540	-0.641409
H	-1.628761	-0.249676	2.336011
O	-0.950273	0.176562	2.896687
F	3.106624	-0.236849	-1.228324
F	2.299133	-2.459460	-0.790646
F	3.128260	-1.137589	1.020807
F	-0.351099	2.241401	-2.174268
F	1.301828	2.742367	-0.493248
F	-1.058057	2.854934	0.037242
F	-3.151319	-0.982602	0.984626
F	-3.072998	-0.186434	-1.306826
F	-2.362689	-2.416575	-0.761251

$[Ru(CO)_3Cl_3]^-$

SCF done: -1816.000711

Ru	-0.012802	0.027902	1.003661
C	-0.107578	1.846824	1.499601
O	-0.165879	2.971027	1.806397
C	-1.868028	-0.310647	1.086944
C	0.064991	0.320799	-0.859556
O	0.113375	0.501597	-2.011218
O	-3.014660	-0.520217	1.138630
Cl	2.429956	0.338092	0.981698
Cl	0.225872	-2.363061	0.465052
Cl	0.009816	-0.452479	3.418716

$[Ru(CO)_2(CO_2H)Cl_3]^{2-}$

SCF done: -1891.805644

Ru	-0.369321	-0.105162	-0.057660
C	-0.441537	1.372001	-1.140062
O	-0.513931	2.326363	-1.830382
C	-1.658287	-0.985282	-1.037871
C	1.167188	-0.925965	-1.208878
O	1.506054	-0.596740	-2.346648
O	-2.487471	-1.532755	-1.679066
Cl	1.400007	1.037487	1.280701
Cl	-0.266947	-2.131251	1.507479
Cl	-2.283555	0.960361	1.406925

	H 1.389350 -2.104847 0.265656<br>O 1.864211 -1.949076 -0.602158<br><br>**$[Ru(CO)_2(CO_2H)Cl_2]^-$**<br>SCF done: -1431.585770<br>Ru 0.000107 -0.059770 0.016390<br>C -0.473735 -1.851499 -0.044246<br>O -0.817006 -2.974173 -0.083479<br>C -0.299104 0.048000 1.849997<br>C 1.957222 -0.550344 0.367399<br>O 2.407814 -1.608901 0.764649<br>O -0.536888 0.118687 3.000018<br>Cl -0.012247 -0.027573 -2.382334<br>Cl 0.368998 2.386486 -0.000154<br>H 2.195693 1.276717 -0.159933<br>O 2.777277 0.489720 0.040957

Atomic coordinates (in Å) of iron complexes, optimised at the RI-BP86/ECP1 level, and corresponding SCF energies (in a.u.).

$Fe(CO)_5$ SCF done: -690.788598

Fe	0.000000	0.000000	0.000000
C	0.000000	0.000000	1.805465
C	0.000000	1.803965	0.000000
C	1.562280	-0.901982	0.000000
C	-1.562279	-0.901983	0.000000
C	0.000000	0.000000	-1.805465
O	0.000000	0.000000	2.967765
O	2.571371	-1.484582	0.000000
O	0.000000	2.969164	0.000000
O	0.000000	0.000000	-2.967765
O	-2.571371	-1.484582	0.000000

$[Fe(CO)_4(COOH)]^-$
SCF done: -766.699729

Fe	0.287836	-0.030774	-0.001515
C	2.042624	-0.317821	0.000233
C	0.066776	-0.789190	-1.599076
C	0.440391	1.747031	-0.006544
C	0.065158	-0.779769	1.600265
O	-0.029269	-1.266674	-2.676809
O	-0.032254	-1.250753	2.680733
O	3.204560	-0.516036	0.001493
O	0.565491	2.917089	-0.009855
C	-1.756506	0.208931	-0.003384
O	-2.431555	1.223973	-0.007099
O	-2.429398	-1.014820	0.000093
H	-1.692541	-1.663803	0.002463

$Fe(CO)_4(PMe_3)$ SCF done: -1038.598916

Fe	-0.700471	0.002044	0.013856
C	-2.481221	0.006257	0.030606
C	-0.620303	1.195401	1.338602
C	-0.637515	-1.743006	0.383262
C	-0.651448	0.553706	-1.682910
O	-3.648715	0.008974	0.041656
O	-0.586755	-2.890930	0.627028
O	-0.557851	1.980939	2.209725
O	-0.609069	0.917003	-2.799157
P	1.555743	-0.002524	-0.005868
C	2.351878	-0.461070	1.598170
H	2.028881	0.246918	2.377273
H	3.451490	-0.442425	1.516164
H	2.022038	-1.470277	1.889935
C	2.336550	1.620145	-0.422381
H	2.016956	2.373735	0.314121
H	1.991972	1.941046	-1.417712
H	3.436946	1.545396	-0.419512
C	2.324598	-1.170649	-1.214732
H	1.996999	-2.196596	-0.985297
H	3.425321	-1.118084	-1.173386
H	1.981660	-0.917174	-2.229938

$[Fe(CO)_3(COOH)(PMe_3)]^-$
SCF done: -1114.478720

Fe	-0.616517	0.022597	-0.189536
C	-2.636524	0.016211	-0.232782
C	-0.634481	1.500836	-1.163336
C	-0.640301	0.231852	1.568273
C	-0.632637	-1.642961	-0.789075
O	-3.460720	0.122181	0.662778
O	-0.608818	0.372083	2.745393
O	-0.568484	2.513731	-1.787743
O	-0.565524	-2.774031	-1.158367
P	1.581783	0.021255	-0.210723
C	2.439518	1.546172	0.431752
H	2.099745	2.409745	-0.162441
H	3.541017	1.471050	0.384914
H	2.122280	1.707755	1.474444
C	2.422246	-0.177703	-1.863893
H	2.088458	0.640425	-2.522555
H	2.088675	-1.129042	-2.309163
H	3.525214	-0.168761	-1.790888
C	2.440945	-1.306556	0.775500
H	2.123224	-1.216508	1.826643
H	3.542391	-1.243274	0.712511
H	2.102586	-2.286892	0.403027
H	-2.304075	-0.201105	-2.061151
O	-3.133376	-0.139389	-1.538143

$Fe(CO)_3(PMe_3)_2$ SCF done: 1386.39830003Fe -0.000330 -0.005456 0.001690

C	0.000471	-0.448372	1.714261
C	-0.000093	-1.275224	-1.230910
C	0.000239	1.699918	-0.471032
O	0.003088	-0.744901	2.860231
O	0.000923	-2.126105	-2.053601
O	0.002008	2.841039	-0.787063
P	-2.217418	0.002580	-0.001514
C	-3.032222	-1.591307	0.474951
H	-2.709202	-2.380325	-0.222566
H	-4.131885	-1.507333	0.454016
H	-2.703098	-1.872682	1.487507
C	-3.015395	0.392907	-1.627789
H	-2.686268	-0.347764	-2.373447
H	-2.680313	1.387787	-1.961281
H	-4.116480	0.379854	-1.555412
C	-3.016240	1.221965	1.141968
H	-2.689662	1.013191	2.173066
H	-4.116912	1.171778	1.086137
H	-2.679036	2.235548	0.873952
P	2.216612	0.002295	-0.001925
C	3.013191	0.438406	-1.617198
H	2.668156	1.437273	-1.927086
H	2.692521	-0.287636	-2.380836
H	4.114125	0.434561	-1.543446
C	3.015080	1.189768	1.174903
H	2.691784	0.949581	2.199991
H	2.674232	2.209747	0.937967
H	4.115623	1.144536	1.114973

$[Fe(CO)_2(COOH)(PMe_3)_2]^-$
SCF done: -1462.250812

Fe	-0.008577	0.473357	0.023929
C	-0.009753	0.187830	1.744438
C	-0.007339	-0.991762	-1.365476
C	-0.008255	2.146348	-0.497934
O	-0.010563	0.045231	2.937294
O	-0.006189	-2.224710	-1.300863
O	-0.008093	3.324536	-0.732341
P	-2.163065	0.179346	-0.019177
C	-2.771204	-1.516575	0.459857
H	-2.209406	-2.248378	-0.142339
H	-3.860106	-1.640708	0.313786
H	-2.516973	-1.683586	1.519275
C	-3.078387	0.447119	-1.626078
H	-2.602759	-0.174409	-2.400573
H	-2.961603	1.503804	-1.919241
H	-4.153411	0.202381	-1.546199
C	-3.185030	1.247662	1.121730
H	-2.820192	1.097721	2.150640
H	-4.264143	1.013358	1.070761
H	-3.021679	2.304313	0.854374
P	2.145949	0.179227	-0.016192
C	3.063251	0.443948	-1.622463
H	2.947237	1.500190	-1.917526
H	2.588390	-0.178646	-2.396561
H	4.138085	0.198965	-1.540821
C	3.166741	1.249500	1.123926
H	2.800864	1.101406	2.152734
H	3.003690	2.305670	0.854509
H	4.245901	1.015071	1.074496

C	3.032247	-1.603840	0.429559
H	2.708446	-1.910681	1.436430
H	4.131909	-1.520027	0.405094
H	2.704898	-2.374259	-0.286215

C	2.752806	-1.516056	0.466516
H	2.497121	-1.681194	1.525872
H	3.841817	-1.641034	0.321983
H	2.191284	-2.248401	-0.135309
O	-0.007821	-0.462898	-2.698538
H	-0.008486	0.502445	-2.490622

$Fe(CO)_2(PMe_3)$2SCF done: -1734.163278

Fe	-0.482867	-0.080975	0.223500
C	-0.376948	1.355316	-0.772393
C	-0.387755	-1.778130	-0.211591
O	-0.296760	2.293920	-1.503477
O	-0.313915	-2.909100	-0.579161
P	1.731019	-0.074678	0.192636
C	2.634764	1.434931	0.803752
H	2.259993	2.311549	0.251831
H	3.723927	1.344199	0.651052
H	2.433811	1.591856	1.875391
C	2.428940	-0.209748	-1.524658
H	2.048892	0.629073	-2.128521
H	2.069948	-1.147850	-1.976649
H	3.532850	-0.197325	-1.524655
C	2.654742	-1.451361	1.037148
H	2.447895	-1.437732	2.117207
H	3.742201	-1.365569	0.871250
H	2.296091	-2.410366	0.631395
P	-0.788567	0.350112	2.394510
C	-2.309929	-0.282608	3.289043
H	-2.336485	-1.382745	3.225604
H	-3.224657	0.112694	2.818991
H	-2.306747	0.015772	4.352259
C	0.461794	-0.224720	3.667791
H	1.452051	0.210432	3.458087
H	0.549523	-1.322465	3.619916
H	0.161349	0.067263	4.689528
C	-0.892668	2.153179	2.880837
H	0.035278	2.660814	2.571669
H	-1.043967	2.297124	3.966066
H	-1.723698	2.624096	2.331304
P	-2.605687	-0.165774	-0.399994
C	-2.811280	-0.319987	-2.240750
H	-2.305875	-1.239969	-2.574523
H	-2.318095	0.537125	-2.725349
H	-3.874284	-0.353089	-2.536886
C	-3.701622	1.301887	-0.062302
H	-3.802111	1.461015	1.023054
H	-4.705220	1.167184	-0.501017
H	-3.228992	2.196257	-0.498480
C	-3.664538	-1.584018	0.173715
H	-3.755698	-1.567836	1.269584
H	-3.171010	-2.525167	-0.115467
H	-4.670234	-1.542216	-0.278256

$[Fe(CO)(COOH)(PMe_3)_3]^-$
SCF done: -1810.006845

Fe	0.017179	-0.036896	0.111677
C	0.017674	-0.204627	1.848614
C	0.017947	0.021049	-1.899984
O	0.018344	-0.348805	3.041161
O	0.018880	0.939918	-2.729095
P	-1.984469	-0.882168	0.033006
C	-3.186851	-0.319471	1.366373
H	-2.720733	-0.484805	2.351363
H	-4.157339	-0.849123	1.321540
H	-3.360075	0.763769	1.252604
C	-2.285671	-2.741303	0.243624
H	-1.812963	-3.063110	1.186806
H	-1.788893	-3.277878	-0.582850
H	-3.359810	-3.016213	0.258883
C	-3.086841	-0.619239	-1.463085
H	-3.218052	0.463627	-1.621438
H	-4.075533	-1.100309	-1.343129
H	-2.572005	-1.027940	-2.346490
P	0.017266	2.139592	0.092730
C	1.429476	3.040719	-0.757538
H	1.466624	2.645315	-1.785234
H	2.374915	2.800085	-0.242031
H	1.295553	4.139870	-0.779737
C	-1.393658	3.041004	-0.759369
H	-2.339819	2.800682	-0.245044
H	-1.429655	2.645546	-1.787088
H	-1.259392	4.140112	-0.781470
C	0.016273	3.071753	1.719961
H	-0.872717	2.779597	2.303062
H	0.016805	4.168629	1.576815
H	0.903976	2.778899	2.304675
P	2.018811	-0.882491	0.033139
C	2.319553	-2.741829	0.242579
H	1.822924	-3.277738	-0.584412
H	1.846490	-3.064153	1.185408
H	3.393637	-3.016931	0.257980
C	3.220849	-0.320898	1.367260
H	3.394511	0.762333	1.254068
H	4.191148	-0.850889	1.322506
H	2.754282	-0.486527	2.351980
C	3.121709	-0.618761	-1.462419
H	3.252970	0.464184	-1.620158
H	2.607240	-1.027027	-2.346236
H	4.110347	-1.099893	-1.342299
H	0.016904	-1.838906	-1.681583
O	0.017518	-1.287877	-2.501673

$Fe(CO)(PMe_3)$4SCF done: -2081.911318

Fe	0.025141	-0.014041	0.278865
C	0.077118	-0.349978	1.972513
O	0.119978	-0.619107	3.142456
P	2.236710	0.072926	0.610662
C	2.987060	-1.485917	1.318445
H	2.401492	-1.801325	2.195382
H	4.038501	-1.325697	1.614472
H	2.949225	-2.290129	0.566252
C	2.797658	1.282371	1.919931
H	2.269557	1.048915	2.857031
H	2.525989	2.307950	1.622520
H	3.888401	1.228202	2.084103
C	3.550167	0.438703	-0.681780
H	3.377619	-0.153224	-1.591564
H	4.549100	0.198359	-0.278492
H	3.531234	1.503523	-0.951956
P	0.065796	-1.706709	-1.155959
C	-1.344752	-2.012994	-2.364920
H	-1.467886	-1.134604	-3.019468
H	-2.289662	-2.168822	-1.819481

$[Fe(COOH)(PMe_3)_4]^-$
SCF done: -2157.736288

Fe	-0.017123	0.005242	-0.165616
C	-0.115120	-1.131576	-1.786054
O	-0.198738	-2.354130	-1.970809
P	-2.194222	0.095989	-0.567174
C	-3.067694	-1.567320	-0.700599
H	-2.471136	-2.223086	-1.352716
H	-4.095258	-1.458772	-1.093494
H	-3.117765	-2.029934	0.299158
C	-2.864283	0.850147	-2.166457
H	-2.369362	0.346242	-3.010529
H	-2.584004	1.917547	-2.193444
H	-3.964479	0.755242	-2.254303
C	-3.472614	0.911700	0.568748
H	-3.266880	0.662029	1.620609
H	-4.489428	0.565321	0.307435
H	-3.439188	2.005473	0.458806
P	-0.148812	-1.306309	1.556338
C	1.229537	-1.384419	2.858804
H	1.356158	-0.383028	3.303022

H	-1.156907	-2.903807	-2.991052
C	1.454217	-1.925099	-2.406358
H	2.410209	-2.091814	-1.883786
H	1.549257	-1.012358	-3.016838
H	1.270565	-2.787484	-3.071960
C	0.141017	-3.434421	-0.419620
H	1.045674	-3.521410	0.202879
H	0.155603	-4.223510	-1.193597
H	-0.726135	-3.591435	0.240839
P	-2.165978	-0.122197	0.685736
C	-2.777060	0.863671	2.151284
H	-2.582016	1.935082	1.994453
H	-2.217876	0.543898	3.043671
H	-3.857807	0.708335	2.315113
C	-2.814966	-1.796055	1.208296
H	-2.747764	-2.511238	0.373451
H	-3.865466	-1.734711	1.541870
H	-2.189005	-2.167844	2.034516
C	-3.505222	0.344712	-0.542519
H	-3.367403	-0.201404	-1.487554
H	-3.460653	1.423648	-0.758879
H	-4.502951	0.111749	-0.131756
P	-0.142159	1.924397	-0.820571
C	1.348528	2.968633	-1.290750
H	1.975021	3.172574	-0.407827
H	1.024638	3.931839	-1.722361
H	1.956095	2.438676	-2.040601
C	-1.122228	3.329423	-0.039413
H	-0.664836	3.580984	0.931522
H	-2.161991	3.019774	-0.680707
H	-1.135837	4.229141	-0.680707
C	-0.927019	1.968218	-2.533326
H	-0.326005	1.331832	-3.204884
H	-0.966056	2.992287	-2.948481
H	-1.948060	1.558097	-2.505093

H	2.181960	-1.666437	2.381136
H	1.009259	-2.112592	3.664261
C	-1.527147	-1.137980	2.849423
H	-2.510497	-1.315393	2.383171
H	-1.518827	-0.107332	3.243881
H	-1.406485	-1.849083	3.690740
C	-0.316780	-3.181861	1.338915
H	-1.196657	-3.390623	0.710103
H	-0.407633	-3.721874	2.302315
H	0.559088	-3.558115	0.786875
P	2.124834	-0.292592	-0.584670
C	2.891220	0.237145	-2.232782
H	2.821058	1.332969	-2.333276
H	2.297829	-0.214009	-3.042372
H	3.951710	-0.070853	-2.313478
C	2.727270	-2.074745	-0.626053
H	2.710235	-2.493168	0.393922
H	3.753967	-2.152935	-1.028296
H	2.022529	-2.647966	-1.248003
C	3.513764	0.387691	0.505865
H	3.363843	0.056234	1.545551
H	3.512378	1.489781	0.491925
H	4.499515	0.032944	0.152229
P	0.243178	2.071069	0.490455
C	-1.158501	3.311605	0.772003
H	-1.772578	3.405172	-0.139308
H	-0.754615	4.308725	1.030889
H	-1.803188	2.967910	1.596251
C	1.270902	3.279026	-0.559202
H	0.755365	3.410961	-1.525672
H	2.260876	2.844664	-0.771856
H	1.408825	4.268500	-0.075712
C	1.097465	2.485263	2.147609
H	0.456280	2.105872	2.962281
H	1.265659	3.570646	2.298588
H	2.063892	1.961622	2.212144
O	-0.082624	-0.361414	-3.002900
H	-0.009145	0.544995	-2.602686

$Fe(CO)_4(PF_3)$ SCF done: -1218.44408588Fe 0.595515 -0.000097 0.000197

C	2.388227	0.001213	-0.000610
C	0.611448	1.781855	-0.235122
C	0.612687	-1.095150	-1.425162
C	0.613462	-0.687433	1.660969
O	3.551436	0.002030	-0.001511
O	0.660705	-1.805620	-2.349787
O	0.658432	2.937882	-0.388081
O	0.662249	-1.133336	2.738376
P	-1.530483	-0.000292	0.000247
F	-2.301339	0.582410	-1.271754
F	-2.301686	0.810175	1.140519
F	-2.301827	-1.392990	0.131097

$[Fe(CO)_3(COOH)(PF_3)]^-$
SCF done: -1294.35567039Fe -0.333013 0.050976 0.001430

C	-2.336559	0.110293	-0.005360
C	-0.505211	-0.823251	-1.533170
C	-0.357688	1.834739	-0.013890
C	-0.510626	-0.791241	1.553451
O	-3.093050	1.079292	-0.019937
O	-0.373118	3.012773	-0.023837
O	-0.628310	-1.393478	-2.560510
O	-0.637467	-1.339144	2.592375
P	1.740621	-0.061531	0.001033
H	-3.884994	-0.949627	0.001925
O	-2.920109	-1.159811	0.008150
F	2.632129	0.646713	-1.164675
F	2.629204	0.512748	1.240332
F	2.506549	-1.496470	-0.079070

$Fe(CO)_3(PF_3)2$ SCF done: -1746.10261419Fe 0.000001 -0.000886 0.001857

C	-0.000124	-0.185579	1.784138
C	0.000348	1.636210	-0.726158
C	-0.000153	-1.454665	-1.044856
O	-0.000376	-0.306089	2.946733
O	0.000592	2.704318	-1.201013
O	-0.000335	-2.404103	-1.726694
P	2.107404	0.000352	-0.002070
P	-2.107414	0.000624	-0.002183
F	2.882733	-1.284281	0.548128
F	2.880884	1.121998	0.833040
F	2.876682	0.166344	-1.393001
F	-2.882846	-1.270608	0.578178
F	-2.876739	0.133597	-1.396600
F	-2.880643	1.141793	0.806319

$[Fe(CO)_2(COOH)(PF_3)_2]^-$
SCF done: -1822.02145550Fe 0.003131 -0.033242 0.515953

C	-0.041547	-1.622189	1.302223
C	0.067044	1.879101	-0.212736
C	-0.048387	0.773589	2.084785
O	-0.054518	-2.655201	1.867021
O	-0.784909	2.454154	-0.876660
O	-0.104886	1.273724	3.152947
P	-1.801169	-0.270752	-0.515025
P	1.712370	-0.432182	-0.566989
O	1.196481	2.620079	0.133426
H	1.757346	2.003294	0.648488
F	1.901815	-0.043055	-2.139600
F	3.149199	0.242114	-0.114610
F	2.311827	-1.942104	-0.701921
F	-2.538933	-1.726942	-0.511773
F	-3.131433	0.567222	-0.096551
F	-1.957672	-0.021684	-2.112495

$Fe(CO)_2(PF_3)_3$SCF done: -2273.76195923Fe 0.000070
-0.039094 0.013892

C	0.000119	-0.152560	1.805171
C	0.000154	-0.030142	-1.779187
O	0.000211	-0.230036	2.967500
O	0.000200	-0.027772	-2.944906
P	-1.861767	-1.001086	-0.018966
P	-0.001303	2.051407	0.044558
P	1.862965	-0.999023	-0.018914
F	2.877467	-0.773137	1.205104
F	2.873592	-0.697841	-1.229212
F	1.956596	-2.603918	-0.066902
F	1.202340	2.846260	-0.669433
F	-0.004857	2.846025	1.437637
F	-1.202911	2.844557	-0.674678
F	-2.876762	-0.775964	1.204781
F	-2.872519	-0.701461	-1.229542
F	-1.953518	-2.606116	-0.066423

$[Fe(CO)(COOH)(PF_3)_3]^-$
SCF done: -2349.68213914Fe -0.000649 0.000047
0.235114

C	-0.092153	0.000234	2.003440
C	0.109796	-0.000949	-1.813519
O	-0.130659	0.000528	3.179593
O	-0.845924	-0.007480	-2.580302
P	0.977384	-1.802304	0.155511
P	-2.076308	-0.008156	-0.014571
P	0.962442	1.810278	0.154550
H	2.025105	0.010751	-1.675738
O	1.372247	0.005806	-2.406136
F	1.196852	-2.711088	1.488675
F	0.487195	-3.032354	-0.794508
F	2.545177	-1.894179	-0.339399
F	2.529332	1.914346	-0.341625
F	0.461873	3.036047	-0.795681
F	1.175758	2.721485	1.487006
F	-2.870660	1.193578	-0.764005
F	-3.022616	-0.009468	1.313767
F	-2.862042	-1.217897	-0.760268

$Fe(CO)(PF_3)_4$SCF done: -2801.41010651Fe 0.000057
-0.013270 0.307542

C	0.000064	-0.080578	2.082372
O	0.000073	-0.128220	3.251504
P	-2.100069	0.012993	0.480960
P	0.000125	1.767788	-0.803184
P	2.100185	0.012692	0.480852
P	-0.000190	-1.771991	-0.831056
F	-3.024745	-0.272076	-0.792464
F	-2.785994	-0.999745	1.507711
F	-2.808188	1.357394	0.984559
F	-1.214563	2.088378	-1.811113
F	0.000247	3.181609	-0.036870
F	1.214848	2.088197	-1.811139
F	2.808540	1.356799	0.984891
F	2.785979	-1.000529	1.507232
F	3.024771	-0.272107	-0.792692
F	-0.001147	-1.712773	-2.440450
F	1.211147	-2.822384	-0.673634
F	-1.211250	-2.822539	-0.672245

$[Fe(COOH)(PF_3)_4]^-$
SCF done: -2877.33685203Fe -0.088745 0.054065
-0.001577

C	-1.801441	1.174839	-0.068044
O	-1.838922	2.395355	-0.159956
P	-0.783539	-0.753260	-1.752846
P	0.909575	1.884959	-0.112289
P	-0.764306	-0.539244	1.839556
P	1.635030	-1.106028	0.055562
O	-3.028100	0.523080	-0.010742
H	-2.844299	-0.436536	0.065959
F	-1.261738	0.525746	2.967142
F	0.123439	-1.442928	2.865259
F	-2.112733	-1.478332	1.965547
F	0.734802	3.033684	1.019498
F	0.760252	2.874267	-1.389465
F	2.543193	1.919917	-0.097658
F	1.533391	-2.725988	0.141498
F	2.723039	-0.953552	1.250231
F	2.714745	-1.080639	-1.155967
F	0.107413	-1.751117	-2.684555
F	-2.114553	-1.721797	-1.753132
F	-1.312907	0.171619	-2.984048

$Fe(CO)_4(py)$SCF done: -825.740758

Fe	0.752630	0.001377	-0.000743
C	0.681465	-0.853396	1.584306
C	0.917637	1.790515	0.010261
C	0.680128	-0.834037	-1.596008
C	2.507891	-0.154019	-0.002562
O	0.645287	-1.363983	-2.640723
O	1.081943	2.952566	0.017433
O	3.671681	-0.263788	-0.003820
O	0.647418	-1.395950	2.622549
C	-2.040316	1.206495	0.009478
C	-2.000939	-1.119045	-0.007377
C	-3.439059	1.220108	0.010464
H	-1.469469	2.136645	0.015866
C	-3.397334	-1.183783	-0.006978
H	-1.386662	-2.021988	-0.014229
C	-4.138855	0.005853	0.002099
H	-3.960852	2.180958	0.017762
H	-3.885277	-2.162225	-0.013737
H	-5.232839	-0.012868	0.002652
N	-1.315117	0.056833	0.000669

$[Fe(CO)_3(COOH)(py)]^-$
SCF done: -901.621139

Fe	-0.918495	0.156276	-0.351861
C	-0.860549	-0.823881	-1.840613
C	-1.112740	1.906805	-0.576229
C	-1.002936	-0.779656	1.161180
C	-2.917439	0.065844	-0.399013
O	-1.019322	-1.436465	2.152410
O	-1.227335	3.077382	-0.767737
O	-3.668271	-0.469832	-1.201626
O	-0.791294	-1.471446	-2.827903
C	1.858975	1.387723	-0.368105
C	1.851844	-0.913803	-0.088199
C	3.255022	1.429093	-0.311514
H	1.271355	2.298641	-0.506115
C	3.246338	-0.951881	-0.017750
H	1.248603	-1.821675	-0.010301
C	3.980929	0.240213	-0.130497
H	3.760243	2.395881	-0.408918
H	3.744463	-1.917033	0.122217
H	5.074717	0.242475	-0.080765
N	1.132039	0.236548	-0.258705
H	-2.736076	1.070089	1.173511
O	-3.517130	0.729767	0.684226

$Fe(CO)_3(bipy)$SCF done: -959.516118

$[Fe(CO)_2(COOH)(bipy)]^-$

Fe	-0.946587	0.037629	-0.021533
C	1.824376	-0.702925	0.004381
C	0.425779	-2.599803	-0.047999
C	2.966387	-1.536166	0.005935
C	1.515994	-3.461495	-0.047659
H	-0.600689	-2.973009	-0.068803
C	2.820222	-2.919601	-0.020051
H	3.961914	-1.085241	0.027645
H	1.346838	-4.541179	-0.068705
H	3.698418	-3.571611	-0.019065
C	1.838345	0.746105	0.029654
C	0.507489	2.680272	0.044363
C	3.008746	1.535407	0.059837
C	1.626560	3.501777	0.074358
H	-0.496741	3.108712	0.037603
C	2.915560	2.923283	0.082542
H	3.987358	1.047377	0.065222
H	1.489996	4.586627	0.091190
H	3.814368	3.545611	0.106005
N	0.554178	-1.241106	-0.022713
N	0.571192	1.310748	0.021568
C	-2.165631	1.306876	-0.016960
C	-1.681681	-0.721964	1.405863
C	-1.642004	-0.672980	-1.493335
O	-2.140381	-1.193260	-2.421479
O	-2.970477	2.161741	-0.013653
O	-2.205005	-1.273104	2.301903

SCF done: -1035.393639

Fe	1.111505	-0.104781	-0.075897
C	-1.752493	-0.312481	-0.009138
C	-0.714210	-2.445219	-0.107988
C	-3.024814	-0.942650	0.078592
C	-1.934636	-3.095648	-0.035680
H	0.228867	-2.986067	-0.230683
C	-3.129175	-2.323999	0.071843
H	-3.920800	-0.317744	0.158464
H	-1.963734	-4.188979	-0.069860
H	-4.107969	-2.810608	0.145517
C	-1.503734	1.091062	-0.021220
C	0.182903	2.754769	-0.043701
C	-2.495581	2.106528	-0.010239
C	-0.752124	3.769497	-0.020456
H	1.253256	2.980386	-0.057662
C	-2.145913	3.447138	-0.009700
H	-3.550681	1.809979	-0.007622
H	-0.410049	4.809810	-0.011552
H	-2.909210	4.231894	-0.005242
N	-0.584046	-1.080458	-0.083220
N	-0.125667	1.405949	-0.048581
C	2.551957	0.866180	-0.217623
C	1.593709	-1.145546	-1.674180
C	1.843114	-1.093834	1.159283
O	2.336202	-1.742367	2.020735
O	3.530821	1.537235	-0.272260
O	1.891597	-2.324090	-1.820986
H	1.216523	0.508248	-2.506407
O	1.472413	-0.375195	-2.849985

$Fe(CO)(H)_2(PMe_3)3$ SCF done: -1621.995996

Fe	-0.027385	0.462510	-0.262450
H	-0.029034	1.952997	0.044963
H	-0.172207	0.375665	1.275627
C	0.114522	0.591010	-2.006446
O	0.208597	0.672673	-3.186903
P	-2.138055	1.059918	-0.223517
P	2.071456	0.942559	0.124326
P	0.015197	-1.740435	-0.067790
C	-3.537692	-0.023236	-0.835101
H	-3.654934	-0.902252	-0.183596
H	-3.306044	-0.373794	-1.854235
H	-4.491234	0.532996	-0.855684
C	-2.496520	2.578650	-1.240408
H	-1.872811	3.408536	-0.873193
H	-3.561483	2.864257	-1.191024
H	-2.216579	2.383639	-2.288161
C	-2.819482	1.574623	1.426985
H	-2.154923	2.342298	1.852966
H	-2.813569	0.707356	2.106285
H	-3.845690	1.973311	1.344788
C	-1.560037	-2.731775	0.104446
H	-1.342694	-3.804947	0.246835
H	-2.175660	-2.609774	-0.800025
H	-2.132473	-2.366078	0.972267
C	0.915511	-2.426585	1.418040
H	1.953996	-2.059161	1.435182
H	0.926693	-3.530536	1.418379
H	0.413885	-2.062702	2.329044
C	0.820029	-2.726572	-1.434842
H	1.862642	-2.395945	-1.562939
H	0.287490	-2.527275	-2.378776
H	0.807476	-3.811369	-1.229482
C	3.470178	-0.153097	-0.463899
H	4.454632	0.285587	-0.224675
H	3.389979	-0.275652	-1.556459
H	3.408187	-1.150761	-0.000423
C	2.565586	1.214065	1.896067
H	3.612798	1.551302	1.989500
H	2.428301	0.278460	2.460989
H	1.890745	1.969934	2.327451
C	2.631497	2.543624	-0.642200
H	3.700886	2.742072	-0.454267
H	2.023943	3.364718	-0.230642
H	2.451045	2.500146	-1.728147

$[Fe(COOH)(H)_2(PMe_3)_3]^-$

SCF done: -1697.827976

Fe	-0.001335	-0.416904	-0.103901
H	-0.065235	-1.952100	-0.357206
H	0.071939	-0.323933	-1.677801
C	-0.241436	-0.561812	1.854975
O	-0.450109	0.278369	2.746553
P	-2.134479	-0.695935	-0.327349
P	2.061573	-1.033433	-0.159087
P	0.241393	1.748383	-0.122967
C	-3.380132	0.292984	0.680531
H	-3.437117	1.326792	0.305343
H	-3.012057	0.323568	1.718891
H	-4.391569	-0.152594	0.650270
C	-2.774612	-2.401696	0.116334
H	-2.282247	-3.140780	-0.536640
H	-3.873138	-2.486679	0.018793
H	-2.471979	-2.605128	1.156415
C	-2.921081	-0.536104	-2.023499
H	-2.394673	-1.223640	-2.704969
H	-2.759563	0.488721	-2.396736
H	-4.004198	-0.763472	-2.021340
C	-1.232037	2.920495	-0.216330
H	-0.919154	3.972779	-0.355199
H	-1.804485	2.841213	0.721606
H	-1.887019	2.628533	-1.054662
C	1.195611	2.485410	-1.565617
H	2.178156	1.991482	-1.640760
H	1.342337	3.578313	-1.471224
H	0.643292	2.260249	-2.492658
C	1.106685	2.604180	1.306990
H	2.172483	2.324863	1.317319
H	0.632664	2.197252	2.216310
H	1.022663	3.706273	1.267078
C	3.505438	0.011071	0.468722
H	4.479854	-0.497408	0.343804
H	3.342135	0.223686	1.538218
H	3.537226	0.973375	-0.069184
C	2.768219	-1.473117	-1.840542
H	3.792626	-1.889566	-1.786657
H	2.764972	-0.568683	-2.470382
H	2.087607	-2.203478	-2.306690
C	2.530413	-2.613412	0.744688
H	3.617068	-2.815629	0.707349
H	1.987792	-3.452043	0.276499
H	2.194158	-2.533135	1.790930
H	-0.129233	-2.404581	1.505208
O	-0.259357	-1.922992	2.354167

$Fe(CO)(H)_2(PF_3)$3SCF done: -2161.59746996Fe - 0.000019 -0.289116 0.017988

H	-0.000082	-1.754732	-0.385241
H	-0.000095	-0.057649	-1.489701
C	0.000050	-0.593625	1.782868
O	0.000102	-0.783062	2.932593
P	-2.000274	-0.786808	-0.295966
P	2.000232	-0.786834	-0.296012
P	0.000019	1.809406	-0.011321
F	3.198777	0.166118	0.188848
F	2.527257	-2.143529	0.370990
F	2.504314	-1.051685	-1.787765
F	1.206068	2.558626	-0.758135
F	0.000047	2.676158	1.338573
F	-1.205971	2.558698	-0.758145
F	-2.527450	-2.143298	0.371324
F	-2.504256	-1.051982	-1.787697
F	-3.198799	0.166357	0.188542

$[Fe(COOH)(H)_2(PF_3)_3]^-$

SCF done: -2237.50256938Fe 0.004440 -0.248664 0.070190

H	-0.001625	-1.572612	0.832922
H	-0.057715	-0.830767	-1.374490
C	0.164262	0.394710	1.972312
O	1.158154	0.868847	2.507735
P	-1.870470	-0.918469	-0.261132
P	1.897463	-0.956489	-0.242061
P	-0.009301	1.729782	-0.512571
H	-1.658962	-0.139086	2.214933
O	-0.967344	0.259566	2.781602
F	2.865944	-0.320024	-1.386193
F	2.029019	-2.486271	-0.774325
F	3.045528	-1.103867	0.893269
F	-0.239563	2.161705	-2.067337
F	1.263639	2.705950	-0.268314
F	-1.120805	2.757020	0.087493
F	-3.032785	-0.985708	0.917635
F	-2.854016	-0.291780	-1.396749
F	-2.087468	-2.460380	-0.717746

Atomic coordinates (in Å) of osmium complexes, optimised at the RI-BP86/ECP1 level, and corresponding SCF energies (in a.u.).

Os(CO)5 SCF done: -657.644692

Os	0.000000	0.000000	0.000000
C	0.000000	0.000000	1.976371
C	0.000000	1.962601	0.000000
C	1.699662	-0.981300	0.000000
C	-1.699662	-0.981300	0.000000
C	0.000000	0.000000	-1.976371
O	0.000000	0.000000	3.138208
O	2.710958	-1.565172	0.000000
O	0.000000	3.130344	0.000000
O	0.000000	0.000000	-3.138208
O	-2.710958	-1.565172	0.000000

[Os(CO)$_4$(COOH)]$^-$
SCF done: -733.561984

Os	0.264724	0.000051	-0.005493
C	2.201931	0.001554	-0.201581
C	0.043931	1.734150	-0.848751
C	0.314077	-0.016239	1.934232
C	0.043688	-1.719810	-0.877326
O	-0.063117	2.799975	-1.352257
O	-0.063661	-2.777162	-1.398333
O	3.372192	0.002588	-0.335514
O	0.353988	-0.026264	3.111812
C	-1.938784	-0.001099	0.173189
O	-2.642606	-0.009294	1.169820
O	-2.589980	0.009272	-1.066891
H	-1.853114	0.014597	-1.714810

Os(CO)$_4$(PMe$_3$) SCF done: -1005.456219

Os	-0.654912	-0.000128	-0.000398
C	-2.601161	-0.001918	-0.006938
C	-0.574325	1.319394	-1.428967
C	-0.587976	0.579175	1.856208
C	-0.573197	-1.897613	-0.426927
O	-3.768510	-0.002078	-0.010633
O	-0.536428	0.929940	2.977726
O	-0.512370	2.116552	-2.291689
O	-0.511623	-3.043698	-0.684340
P	1.776420	0.001221	0.003326
C	2.548533	1.617666	0.450482
H	2.210254	2.387026	-0.260971
H	3.649329	1.553538	0.430405
H	2.215036	1.910585	1.458178
C	2.544313	-0.419820	-1.622194
H	2.204181	0.304649	-2.378558
H	2.207535	-1.421866	-1.930476
H	3.645342	-0.402445	-1.561770
C	2.546393	-1.196218	1.179492
H	2.208909	-0.964983	2.201903
H	3.647200	-1.148886	1.134025
H	2.210746	-2.214704	0.929006

[Os(CO)$_3$(COOH)(PMe$_3$)]$^-$
SCF done: -1081.340703

Os	-0.401002	0.033869	0.001114
C	-2.531899	0.164970	-0.016315
C	-0.505915	-1.065702	-1.576622
C	-0.334685	1.960256	-0.126320
C	-0.519887	-0.830091	1.718011
O	-3.267683	1.155436	-0.043512
O	-0.246327	3.143149	-0.205889
O	-0.547204	-1.746666	-2.552259
O	-0.576239	-1.365070	2.780023
P	1.976624	-0.062539	0.003153
C	2.862970	0.880924	-1.335318
H	2.512165	0.510305	-2.311693
H	3.962065	0.782493	-1.270937
H	2.578896	1.942586	-1.257486
C	2.750857	-1.746174	-0.176358
H	2.388927	-2.196891	-1.114373
H	2.404293	-2.379291	0.656060
H	3.855509	-1.709136	-0.181792
C	2.851667	0.570958	1.520032
H	2.568659	1.625382	1.669260
H	3.951170	0.487051	1.444736
H	2.493845	0.000452	2.392091
H	-4.127210	-0.818367	-0.005375
O	-3.175968	-1.084540	0.007790

Os(CO)$_3$(PMe$_3$)2 SCF done: -1353.25748392Os   0.000011
-0.001308   -0.001944

C	-0.000639	-0.682714	1.807232
C	-0.000615	-1.229336	-1.491806
C	0.000746	1.904472	-0.315571
O	-0.000010	-1.104962	2.914082
O	-0.001714	-1.979434	-2.408882
O	0.001394	3.073099	-0.510323
P	-2.387022	0.001838	0.000898
C	-3.182820	-1.628746	0.364812
H	-2.856432	-2.361290	-0.389664
H	-4.283534	-1.553700	0.355501
H	-2.844718	-1.980532	1.352005
C	-3.185528	0.503330	-1.590335
H	-2.853289	-0.180472	-2.386713
H	-2.855930	1.520537	-1.853275
H	-4.285746	0.478086	-1.515415
C	-3.167996	1.135901	1.236197
H	-2.820894	0.858057	2.243317
H	-4.269266	1.087000	1.194285
H	-2.835901	2.165803	1.032912
P	2.387092	0.001427	0.000185
C	3.182929	0.450962	-1.607975
H	2.850127	1.458460	-1.903461
H	2.850892	-0.259481	-2.381272
H	4.283407	0.431213	-1.534185
C	3.168891	1.174413	1.198121
H	2.823659	0.926400	2.214069
H	2.836509	2.198338	0.965221
H	4.270173	1.124077	1.157168

[Os(CO)$_2$(COOH)(PMe$_3$)$_2$]$^-$
SCF done: -1429.113999

Os	0.001884	-0.260342	0.062679
C	-0.001777	-1.542668	-1.348232
C	0.015241	1.843615	-0.523937
C	0.006321	-0.713039	1.917395
O	-0.002289	-2.407360	-2.183675
O	0.081868	2.405745	-1.618826
O	0.007662	-1.085283	3.061847
P	-2.327493	-0.054427	-0.084777
C	-2.948859	0.501303	-1.746473
H	-2.401505	1.419302	-2.013334
H	-4.039899	0.678859	-1.759334
H	-2.685732	-0.271294	-2.486654
C	-3.152680	1.168827	1.051509
H	-2.663153	2.144978	0.907941
H	-2.979482	0.849237	2.092198
H	-4.238535	1.254924	0.863515
C	-3.360674	-1.575238	0.214175
H	-3.030224	-2.356452	-0.488755
H	-4.442856	-1.388365	0.086659
H	-3.161654	-1.935106	1.236540
P	2.330918	-0.055667	-0.083889
C	3.143101	1.187514	1.038943
H	2.960135	0.883834	2.082623
H	2.654640	2.161205	0.877864
H	4.230554	1.272553	0.859794
C	3.366417	-1.568190	0.246495
H	3.040885	-2.362694	-0.443804
H	3.164614	-1.910521	1.274351
H	4.448588	-1.380865	0.119633

C	3.184789	-1.615636	0.417902
H	2.853825	-1.931267	1.419939
H	4.285481	-1.540974	0.398133
H	2.853880	-2.375319	-0.307536

C	2.950839	0.473585	-1.753610
H	2.717329	-0.326610	-2.474357
H	4.036249	0.682541	-1.762557
H	2.375450	1.366831	-2.046002
O	-0.065438	2.728616	0.600451
H	-0.087561	2.069444	1.335827

Os(CO)$_2$(PMe$_3$)3 SCF done: -1701.029248

Os	-0.002496	-0.318437	0.023852
C	-0.004742	-1.108600	-1.721664
C	-0.008541	-1.045755	1.799653
O	-0.006303	-1.665615	-2.775796
O	-0.013096	-1.562308	2.873136
P	2.351516	-0.618831	0.001342
C	3.324727	0.196690	-1.355583
H	2.901667	-0.115040	-2.323761
H	4.391698	-0.081926	-1.314346
H	3.234980	1.291757	-1.278856
C	2.865693	-2.388750	-0.216311
H	2.442999	-2.768092	-1.159824
H	2.441718	-2.982471	0.608524
H	3.964313	-2.499637	-0.228985
C	3.307886	-0.155188	1.522878
H	3.204769	0.923310	1.714541
H	4.376493	-0.410568	1.420575
H	2.881261	-0.697487	2.381378
P	0.013592	2.063577	-0.010462
C	-1.399966	3.010357	0.766465
H	-1.479535	2.733573	1.830235
H	-2.348251	2.752338	0.268701
H	-1.247711	4.101484	0.686626
C	1.410146	2.991077	0.819692
H	2.372620	2.723685	0.355015
H	1.449117	2.710522	1.884711
H	1.273652	4.084157	0.737513
C	0.052923	2.898341	-1.682595
H	0.950798	2.564684	-2.227136
H	0.057503	4.001259	-1.607878
H	-0.824124	2.573545	-2.264735
P	-2.359480	-0.596984	-0.002116
C	-2.885022	-2.366509	-0.195184
H	-2.466005	-2.950764	0.638903
H	-2.463322	-2.762273	-1.132350
H	-3.984335	-2.470494	-0.207918
C	-3.323825	0.204012	-1.374244
H	-3.233307	1.299992	-1.312490
H	-4.391422	-0.072376	-1.333470
H	-2.897532	-0.121613	-2.336370
C	-3.319186	-0.106122	1.509018
H	-3.214670	0.975108	1.684069
H	-2.896947	-0.636239	2.377291
H	-4.388039	-0.360613	1.406796

[Os(CO)(COOH)(PMe$_3$)$_3$]$^-$
SCF done: -1776.881939

Os	0.004958	-0.055000	-0.165904
C	-0.002367	-0.174031	-2.068379
C	0.012762	0.075146	2.007351
O	-0.008339	-0.263193	-3.263217
O	0.100900	1.023616	2.797196
P	1.998992	-1.247059	-0.033612
C	3.294712	-0.856206	-1.335526
H	2.838013	-0.974468	-2.331791
H	4.189458	-1.503877	-1.261247
H	3.593054	0.199063	-1.225105
C	2.036503	-3.126715	-0.235620
H	1.526528	-3.381416	-1.179625
H	1.461776	-3.580805	0.589125
H	3.061457	-3.550375	-0.243682
C	3.068248	-1.122747	1.497041
H	3.374126	-0.072199	1.626988
H	3.965438	-1.766526	1.431843
H	2.460956	-1.406371	2.370798
P	0.224254	2.263182	-0.103156
C	-1.100117	3.264562	0.763498
H	-1.164715	2.854869	1.784274
H	-2.062347	3.108448	0.248351
H	-0.870254	4.347367	0.804437
C	1.709419	2.998622	0.769325
H	2.627374	2.664534	0.258073
H	1.691804	2.584477	1.790197
H	1.686643	4.105341	0.809745
C	0.315167	3.188099	-1.724585
H	1.175268	2.814737	-2.303595
H	0.418134	4.279175	-1.574385
H	-0.597323	2.982525	-2.307274
P	-2.177856	-0.851704	-0.042295
C	-2.566547	-2.691024	-0.245134
H	-2.090648	-3.244539	0.581821
H	-2.109523	-3.037291	-1.187111
H	-3.652748	-2.914843	-0.257593
C	-3.371832	-0.225226	-1.349423
H	-3.467322	0.867327	-1.239863
H	-4.372511	-0.693476	-1.279081
H	-2.941318	-0.427507	-2.343829
C	-3.211110	-0.528333	1.483965
H	-3.314974	0.561004	1.613028
H	-2.671431	-0.920498	2.360122
H	-4.212881	-0.992365	1.414826
H	-0.163095	-1.785859	1.858473
O	-0.109487	-1.202111	2.654101

Os(CO)(PMe$_3$)4 SCF done: -2048.785074

Os	-0.023627	-0.012183	-0.271283
C	-0.091447	-0.272144	-2.143503
O	-0.145344	-0.456486	-3.327048
P	-2.385968	0.061989	-0.538315
C	-3.122314	-1.484210	-1.274166
H	-2.578801	-1.730598	-2.199218
H	-4.196198	-1.356072	-1.497102
H	-2.997837	-2.322683	-0.570051
C	-2.978763	1.313407	-1.783862
H	-2.442857	1.138809	-2.729506
H	-2.727398	2.327706	-1.435705
H	-4.068160	1.242185	-1.951094
C	-3.630838	0.365897	0.822643
H	-3.489794	-0.349359	1.646458
H	-4.654729	0.258405	0.424612
H	-3.514403	1.383248	1.222048
P	-0.038486	-1.820386	1.247840
C	1.372656	-2.085155	2.455596
H	1.454591	-1.204598	3.113334
H	2.322733	-2.194547	1.907677

[Os(COOH)(PMe$_3$)$_4$]$^-$
SCF done: -2124.623857

Os	0.030012	-0.136922	-0.076162
C	0.315522	-2.245654	-0.285213
O	0.388985	-3.198719	0.499918
P	-1.998331	-0.444285	-1.175703
C	-2.552690	-2.174935	-1.650770
H	-1.814148	-2.635882	-2.321934
H	-3.550961	-2.156513	-2.126377
H	-2.591531	-2.790596	-0.737495
C	-2.418020	0.405202	-2.815610
H	-1.716103	0.029766	-3.578771
H	-2.254306	1.492022	-2.719596
H	-3.460764	0.225552	-3.148755
C	-3.613501	0.002227	-0.283812
H	-3.689321	-0.637553	0.611997
H	-4.511419	-0.152701	-0.914529
H	-3.586270	1.049575	0.053210
P	-0.343913	-0.376837	2.190406
C	1.101823	-0.393990	3.408079
H	1.654635	0.556828	3.320520

H	1.220022	-2.988707	3.073474
C	-1.442574	-2.050897	2.468410
H	-2.384778	-2.209034	1.918963
H	-1.547333	-1.140767	3.080820
H	-1.274125	-2.920917	3.128409
C	-0.076418	-3.537137	0.491627
H	-0.976048	-3.623826	-0.139010
H	-0.081008	-4.340381	1.251893
H	0.796661	-3.661730	-0.168457
P	2.314931	-0.119259	-0.644639
C	2.921813	0.895629	-2.082801
H	2.687848	1.956571	-1.907161
H	2.382948	0.578131	-2.988537
H	4.008816	0.775388	-2.235108
C	2.984559	-1.784646	-1.150409
H	2.873417	-2.505666	-0.324762
H	4.049813	-1.726375	-1.435130
H	2.393881	-2.148760	-2.005717
C	3.590421	0.366810	0.631716
H	3.453900	-0.212277	1.557499
H	3.486462	1.435718	0.875721
H	4.609389	0.192917	0.244553
P	0.145031	2.008648	0.959687
C	-1.364683	3.019130	1.423443
H	-1.985582	3.224383	0.536713
H	-1.062646	3.980828	1.874445
H	-1.968933	2.464720	2.158626
C	1.131730	3.410769	0.192193
H	0.674138	3.678307	-0.773711
H	2.164362	3.081685	-0.005586
H	1.164681	4.302164	0.845239
C	0.933010	2.036358	2.668836
H	0.330153	1.400292	3.338788
H	0.993131	3.056235	3.092788
H	1.946542	1.608669	2.620739

H	1.782336	-1.212270	3.119201
H	0.786594	-0.538100	4.461785
C	-1.418854	0.843488	3.151617
H	-2.430622	0.852571	2.710938
H	-1.007447	1.864016	3.075350
H	-1.494694	0.572325	4.222987
C	-1.186946	-1.935004	2.795618
H	-2.225573	-1.936135	2.423250
H	-1.191368	-2.012688	3.899665
H	-0.653963	-2.781063	2.332159
P	2.296169	-0.117680	-0.647689
C	2.900704	-0.220440	-2.445958
H	2.472443	0.625047	-3.010090
H	2.509054	-1.155182	-2.879116
H	4.006392	-0.211445	-2.540771
C	3.347236	-1.524783	0.017710
H	3.303298	-1.501083	1.118569
H	4.398807	-1.447447	-0.317073
H	2.918829	-2.481114	-0.316191
C	3.469956	1.270763	-0.141106
H	3.442661	1.380457	0.955239
H	3.165801	2.227479	-0.596962
H	4.507066	1.049517	-0.456393
P	-0.140147	2.207819	-0.108673
C	-1.792811	3.084359	0.138066
H	-2.481015	2.805575	-0.675826
H	-1.676708	4.184322	0.140131
H	-2.240782	2.764504	1.092859
C	0.344080	3.138169	-1.675914
H	-0.326728	2.822367	-2.491816
H	1.367907	2.849947	-1.963022
H	0.288530	4.238284	-1.559425
C	0.826508	3.281435	1.110212
H	0.545869	2.998914	2.138274
H	0.625061	4.359745	0.963970
H	1.905144	3.099150	0.997340
O	0.533722	-2.612871	-1.654047
H	0.416032	-1.719653	-2.074606

$Os(CO)_4(PF_3)$ SCF done: -1185.298448

Os	0.564790	-0.000219	-0.001363
C	2.521746	0.007267	0.001840
C	0.574077	-1.388231	1.381726
C	0.566389	1.893933	0.501471
C	0.574856	-0.513723	-1.892569
O	3.684588	0.012710	0.004360
O	0.589443	3.022794	0.801508
O	0.602442	-2.215660	2.205978
O	0.603784	-0.819646	-3.019665
P	-1.744364	-0.000256	0.002151
F	-2.509957	-0.993616	0.987009
F	-2.515190	-0.353339	-1.348439
F	-2.506393	1.350419	0.374116

$[Os(CO)_3(COOH)(PF_3)]^-$
SCF done: -1261.217412

Os	-0.315383	0.029261	-0.000062
C	-2.459366	0.158641	-0.000187
C	-0.511504	-0.955159	-1.655079
C	-0.342339	1.974449	0.000008
C	-0.511700	-0.955261	1.654869
O	-3.182457	1.154281	-0.000021
O	-0.351924	3.153538	0.000059
O	-0.633332	-1.554552	-2.666138
O	-0.633661	-1.554712	2.665877
P	1.945929	-0.047741	0.000085
H	-4.044527	-0.841166	-0.000147
O	-3.088731	-1.089352	-0.000180
F	2.838801	1.309707	0.000323
F	2.760672	-0.775854	1.202660
F	2.760884	-0.775566	-1.202522

$Os(CO)_3(PF_3)2$ SCF done: -1712.952907

Os	0.000007	-0.003948	0.002608
C	0.000007	-1.329289	-1.436775
C	0.000038	1.908729	-0.411098
C	-0.000007	-0.604877	1.864975
O	-0.000023	-2.121385	-2.296232
O	0.000033	3.051079	-0.658537
O	-0.000047	-0.963939	2.977188
P	-2.286938	0.004420	-0.002938
P	2.286952	0.004366	-0.002906
F	-3.062314	-0.412011	1.327058
F	-3.056907	-0.935663	-1.035767
F	-3.045580	1.373808	-0.307623
F	3.056912	-0.935732	-1.035727
F	3.062299	-0.412087	1.327099
F	3.045631	1.373736	-0.307577

$[Os(CO)_2(COOH)(PF_3)_2]^-$
SCF done: -1788.879961

Os	0.007213	-0.008251	0.517025
C	-0.061492	-1.724424	1.425777
C	0.048229	1.993203	-0.408874
C	0.017956	0.983430	2.167866
O	-0.087680	-2.747165	2.006052
O	-0.833241	2.541771	-1.055356
O	0.021914	1.572619	3.192191
P	-1.937614	-0.318939	-0.586530
P	1.840989	-0.491422	-0.666582
O	1.217514	2.726468	-0.190066
H	1.795278	2.132042	0.332648
F	1.952341	-0.304139	-2.280156
F	3.250370	0.300892	-0.375622
F	2.490203	-1.980755	-0.614115
F	-2.629436	-1.790629	-0.549450
F	-3.283043	0.500362	-0.192612
F	-2.089316	-0.085491	-2.181764

$Os(CO)_2(PF_3)_3$ SCF done: -2240.614600

Os	-0.021821	-0.023578	0.000015
C	-0.028811	-0.022725	1.970100
C	-0.028466	-0.022825	-1.970070
O	-0.033635	-0.019731	3.133294
O	-0.033046	-0.019894	-3.133267
P	-2.237238	-0.545745	-0.000161
P	0.630861	2.144448	-0.000001
P	1.616696	-1.595057	0.000166
F	2.649756	-1.611083	1.226472
F	2.650663	-1.610355	-1.225394
F	1.292208	-3.168980	-0.000472
F	1.520064	2.669557	-1.226540
F	1.519352	2.669797	1.226948
F	-0.433549	3.348709	-0.000448
F	-3.136263	-0.036320	1.226085
F	-3.136037	-0.036458	-1.226630
F	-2.752292	-2.068543	-0.000118

$[Os(CO)(COOH)(PF_3)_3]^-$
SCF done: -2316.539289

Os	-0.008454	-0.000042	0.237957
C	-0.122065	-0.000098	2.175912
C	0.062218	0.000071	-1.968613
O	-0.171147	-0.000197	3.350207
O	-0.892207	-0.000455	-2.734831
P	1.077428	-1.941981	0.126695
P	-2.247566	-0.000603	-0.030307
P	1.076544	1.942394	0.126758
H	1.968601	0.001091	-1.809019
O	1.331497	0.000735	-2.553245
F	1.376064	-2.790334	1.479211
F	0.547074	-3.199464	-0.758203
F	2.604508	-2.033551	-0.461250
F	2.603624	2.034659	-0.461057
F	0.545693	3.199639	-0.758171
F	1.374706	2.790862	1.479308
F	-3.030819	1.196713	-0.789587
F	-3.179504	-0.000853	1.301357
F	-3.030170	-1.198359	-0.789560

$Os(CO)(PF_3)_4$ SCF done: -2768.261688

Os	0.000004	-0.033152	0.341067
C	0.000052	-0.310006	2.267414
O	0.000001	-0.487120	3.423756
P	-2.282251	0.014047	0.475287
P	0.000029	1.940039	-0.793608
P	2.282266	0.013974	0.475187
P	-0.000048	-1.844450	-1.018414
F	-3.166077	-0.513932	-0.743110
F	-2.974694	-0.797181	1.658154
F	-3.007816	1.419519	0.690993
F	-1.222859	2.262473	-1.785700
F	0.000037	3.352118	-0.025316
F	1.222932	2.262446	-1.785692
F	3.007878	1.419413	0.690952
F	2.974742	-0.797351	1.657969
F	3.166019	-0.513949	-0.743287
F	-0.000187	-1.732794	-2.624216
F	1.218116	-2.885978	-0.898690
F	-1.218145	-2.886036	-0.898496

$[Os(COOH)(PF_3)_4]^-$
SCF done: -2844.195430

Os	-0.091703	0.055708	0.001795
C	-1.904071	1.282290	0.007838
O	-1.960501	2.503660	0.000557
P	-0.860359	-0.740621	-1.928534
P	0.979271	2.036533	-0.020839
P	-0.829691	-0.713077	1.955175
P	1.799668	-1.182329	-0.004305
O	-3.116441	0.597736	0.021602
H	-2.894925	-0.356879	0.025829
F	-1.262437	0.279736	3.167887
F	0.064708	-1.722142	2.862539
F	-2.206605	-1.598664	2.047772
F	0.807298	3.119902	1.170209
F	0.786508	3.103596	-1.223341
F	2.606381	2.062106	-0.035091
F	1.716042	-2.800039	0.008300
F	2.885098	-1.064582	1.190937
F	2.865851	-1.082348	-1.218339
F	0.019165	-1.763427	-2.835038
F	-2.239134	-1.626337	-1.986750
F	-1.311469	0.234795	-3.148683

$Os(CO)_4(py)$ SCF done: -792.600221

Os	0.842816	-0.009728	0.000057
C	0.728113	-0.935960	1.717379
C	0.946678	1.935757	-0.005992
C	0.729346	-0.945719	-1.712013
C	2.749612	-0.138138	0.001021
O	0.672343	-1.501404	-2.745622
O	1.052290	3.107503	-0.009689
O	3.915780	-0.223956	0.001659
O	0.670187	-1.485831	2.754036
C	-2.095988	1.208689	-0.001056
C	-2.074658	-1.121652	0.000438
C	-3.493658	1.232514	-0.000907
H	-1.515593	2.132820	-0.001690
C	-3.471636	-1.173184	0.000617
H	-1.465108	-2.027892	0.001059
C	-4.202211	0.022876	-0.000058
H	-4.008255	2.197135	-0.001438
H	-3.968406	-2.147092	0.001305
H	-5.296297	0.013055	0.000090
N	-1.381541	0.050901	-0.000426

$[Os(CO)_3(COOH)(py)]^-$
SCF done: -868.485605

Os	0.632230	0.001112	0.022413
C	0.602262	-0.998550	1.671706
C	0.738889	1.920492	0.115343
C	0.626686	-1.031026	-1.607037
C	2.744578	-0.077726	0.041386
O	0.575211	-1.673142	-2.609278
O	0.758256	3.112995	0.180313
O	3.505825	-0.590704	0.851379
O	0.551583	-1.613201	2.685457
C	-2.347862	1.206021	-0.022715
C	-2.331633	-1.115972	-0.000559
C	-3.745486	1.235243	-0.059855
H	-1.757240	2.125727	-0.015349
C	-3.728514	-1.167595	-0.040628
H	-1.718546	-2.020917	0.027689
C	-4.462608	0.028061	-0.071329
H	-4.257334	2.202782	-0.080251
H	-4.226364	-2.142590	-0.045445
H	-5.557139	0.020050	-0.102323
N	-1.626607	0.051022	0.006108
H	2.573183	0.895545	-1.570132
O	3.344287	0.562523	-1.061714

$Os(CO)_3(bipy)$ SCF done: -926.371460

$[Os(CO)_2(COOH)(bipy)]^-$

Os	-1.060801	0.006837	-0.114884
C	1.869313	-0.717959	-0.093984
C	0.539324	-2.670348	-0.270830
C	3.035352	-1.522475	-0.076945
C	1.656013	-3.487505	-0.265870
H	-0.467508	-3.086793	-0.340099
C	2.943939	-2.903968	-0.167125
H	4.013241	-1.041839	0.008831
H	1.524645	-4.570204	-0.340174
H	3.843685	-3.525262	-0.158352
C	1.869387	0.722435	-0.000748
C	0.539635	2.681498	0.075310
C	3.035498	1.517885	0.120142
C	1.656404	3.491063	0.185748
H	-0.467153	3.103506	0.060171
C	2.944246	2.899494	0.208774
H	4.013310	1.030064	0.143580
H	1.525146	4.574325	0.251554
H	3.844054	3.514342	0.297853
N	0.607648	-1.298657	-0.198003
N	0.607795	1.311859	-0.029187
C	-2.255030	1.383346	-0.694420
C	-1.584833	-0.112482	1.726864
C	-2.255112	-1.283640	-0.866597
O	-2.953878	-2.113901	-1.322152
O	-2.953580	2.265380	-1.039634
O	-2.053680	-0.182416	2.802194

SCF done: -1002.263242

Os	0.994118	-0.114182	-0.318757
C	-1.844859	0.895897	-0.120369
C	-0.299051	2.717450	-0.226021
C	-2.909024	1.842802	-0.014759
C	-1.308849	3.649646	-0.132884
H	0.747822	3.023399	-0.300199
C	-2.667746	3.201113	-0.025099
H	-3.933492	1.466747	0.076945
H	-1.057609	4.714468	-0.138595
H	-3.491501	3.918178	0.054378
C	-1.996719	-0.512333	-0.127680
C	-0.868437	-2.620756	-0.262597
C	-3.237175	-1.216128	-0.042927
C	-2.053100	-3.318786	-0.189160
H	0.090726	-3.139610	-0.341863
C	-3.288195	-2.594432	-0.076111
H	-4.160109	-0.633951	0.050859
H	-2.033234	-4.412393	-0.215405
H	-4.245224	-3.122550	-0.012390
N	-0.503833	1.350592	-0.243082
N	-0.778031	-1.239810	-0.252829
C	2.118034	-1.579092	-0.737160
C	1.504554	-0.150012	1.710525
C	2.386062	1.089151	-0.753540
O	3.217801	1.875343	-1.065007
O	2.780193	-2.519376	-1.029683
O	2.592702	-0.052627	2.255782
H	-0.390557	-0.393031	1.964768
O	0.389027	-0.304350	2.552812

$Os(CO)(H)_2(PMe_3)_3$ SCF done: -1588.884329

Os	-0.056465	-0.387173	-0.044693
H	-0.159995	-2.016759	-0.406711
H	-0.039187	-0.224024	-1.746293
C	-0.088339	-0.671209	1.853670
O	-0.109050	-0.856261	3.025381
P	-2.380411	-0.659149	-0.301434
P	2.219208	-0.932626	-0.269443
P	0.185378	1.981720	-0.036695
C	-3.626315	0.478918	0.492889
H	-3.567994	1.479732	0.039322
H	-3.397335	0.568911	1.567087
H	-4.653008	0.091554	0.372421
C	-2.993861	-2.288823	0.347995
H	-2.451221	-3.098589	-0.163265
H	-4.080498	-2.408138	0.193797
H	-2.764632	-2.353503	1.423342
C	-2.999094	-0.707885	-2.049799
H	-2.427515	-1.474815	-2.594719
H	-2.803640	0.263294	-2.530690
H	-4.077573	-0.937619	-2.097210
C	-1.288049	3.113475	-0.235766
H	-0.976300	4.171881	-0.278214
H	-1.972610	2.980055	0.616369
H	-1.826718	2.863653	-1.164121
C	1.258564	2.696340	-1.384094
H	2.269953	2.262547	-1.330434
H	1.335098	3.795134	-1.308815
H	0.824722	2.421818	-2.358723
C	0.943293	2.772258	1.473641
H	1.933061	2.330099	1.666368
H	0.303318	2.552418	2.343508
H	1.047950	3.865920	1.363387
C	3.555742	0.058759	0.575149
H	4.555700	-0.371265	0.390393
H	3.362083	0.065980	1.660156
H	3.545588	1.100141	0.215450
C	2.868108	-1.013136	-2.005828
H	3.920611	-1.344148	-2.034575
H	2.776224	-0.021857	-2.476084
H	2.238673	-1.714626	-2.574735
C	2.652225	-2.627226	0.356847
H	3.721043	-2.857789	0.204441
H	2.030821	-3.366816	-0.170866
H	2.412614	-2.685897	1.430341

$[Os(COOH)(H)_2(PMe_3)_3]^-$

SCF done: -1664.722616

Os	-0.000021	-0.407023	-0.187431
P	-2.267403	-0.737342	-0.430756
P	2.267281	-0.738466	-0.429967
P	0.000558	1.919549	-0.140939
H	-0.000467	-2.110812	-0.114510
H	0.000239	-0.531400	-1.917498
C	-0.000407	-0.384768	1.952852
O	-0.000840	-1.689575	2.550874
H	-0.000989	-2.238066	1.731966
O	-0.000249	0.539931	2.785687
C	0.000610	2.961675	-1.705815
H	-0.888160	2.701781	-2.304266
H	0.000910	4.048951	-1.498440
H	0.889091	2.701339	-2.304501
C	1.399705	2.747701	0.788206
H	2.365674	2.520679	0.306720
H	1.280539	3.845368	0.855960
H	1.377075	2.297634	1.795334
C	-1.397919	2.748393	0.788604
H	-1.278252	3.846013	0.856228
H	-2.364152	2.521753	0.307472
H	-1.375142	2.298414	1.795766
C	-3.194820	0.219150	-1.749674
H	-4.254709	-0.086195	-1.833098
H	-3.146671	1.297351	-1.524499
H	-2.676308	0.050038	-2.706958
C	-3.454469	-0.521334	1.005754
H	-4.496921	-0.788335	0.748664
H	-3.100567	-1.154614	1.835283
H	-3.415996	0.523974	1.352114
C	-2.749181	-2.466813	-0.948782
H	-3.837015	-2.571828	-1.119765
H	-2.192952	-2.711007	-1.867294
H	-2.423926	-3.166247	-0.162431
C	2.748371	-2.468078	-0.948168
H	2.192352	-2.711800	-1.866933
H	3.836213	-2.573611	-1.118780
H	2.422477	-3.167511	-0.162081
C	3.453955	-0.523354	1.007006
H	3.099425	-1.156594	1.836298
H	4.496352	-0.790871	0.750232
H	3.415931	0.521911	1.353546
C	3.195656	0.217835	-1.748345
H	3.147984	1.296009	-1.522938
H	4.255417	-0.088041	-1.831469
H	2.677385	0.049210	-2.705845

$Os(CO)(H)_2(PF_3)_3$ SCF done: -2128.468033

Os	-0.000003	-0.384766	0.022876
H	-0.000008	-2.042578	-0.188509
H	0.000007	-0.365607	-1.666154
C	-0.000017	-0.643583	1.971719
O	-0.000030	-0.833911	3.120494
P	-2.210790	-0.748992	-0.311721
P	2.210785	-0.749006	-0.311693
P	0.000007	1.907349	-0.036859
F	3.202708	0.442552	-0.695822
F	3.074175	-1.415491	0.855260
F	2.587824	-1.752829	-1.487135
F	1.207362	2.657615	-0.773048
F	0.000041	2.762510	1.320285
F	-1.207367	2.657633	-0.772999
F	-3.074200	-1.415467	0.855222
F	-2.587819	-1.752817	-1.487165
F	-3.202700	0.442570	-0.695868

$[Os(COOH)(H)_2(PF_3)_3]^-$
SCF done: -2204.369049

Os	-0.016624	-0.348305	-0.052287
H	-0.030641	-1.964646	0.420911
H	-0.010463	-0.881979	-1.671691
C	0.152103	0.078262	2.085855
O	0.987299	0.781801	2.643411
P	-2.180872	-0.739151	-0.286965
P	2.155804	-0.824394	-0.275395
P	-0.010786	1.843467	-0.525846
H	-1.369963	-1.043810	2.341429
O	-0.776603	-0.545285	2.937079
F	3.124231	0.088720	-1.193156
F	2.517606	-2.229150	-0.979550
F	3.176072	-0.999525	0.961432
F	-0.251621	2.406019	-2.034475
F	1.282855	2.766583	-0.223198
F	-1.109504	2.820625	0.160194
F	-3.146664	-1.094531	0.984950
F	-3.198159	0.304366	-0.991282
F	-2.633207	-2.028783	-1.145195
